# Developmentally dynamic chromatin state at loci regulating organ crosstalk by remote sensing and signaling

**DOI:** 10.1186/s13072-025-00648-9

**Published:** 2025-12-03

**Authors:** Aditya Parmar, Sanjay K. Nigam, Kun Cai, Kian Falah, Vladimir S. Ermakov, Kelly Wang, Cole J. Ferguson

**Affiliations:** 1 Department of Pathology, University of California San Diego, 9500 Gilman Dr., San Diego, CA 92093 USA; 2 Department of Pediatrics, University of California San Diego, 9500 Gilman Dr., San Diego, CA 92093 USA

**Keywords:** Chromatin, Epigenomics, Epigenetics, Remote sensing and signaling theory, ADME genes, SLC transporters, ABC transporters

## Abstract

**Background:**

Interorgan communication, metabolite regulation and drug handling require fine-tuned small molecule transport across membranes. The Remote Sensing and Signaling (RSS) theory, which has found applicability in chronic kidney disease and uric acid disorders, emphasizes the central role of solute carrier (SLC) and ATP-binding cassette (ABC) transporters, enzymes and transcription factors in organ crosstalk. Based on prior network biology studies, ~ 1000 protein-coding genes are predicted to mediate RSS. This gene set largely overlaps with genes that are important for absorption, digestion, metabolism and excretion (ADME) of small molecules. However, it is not known how epigenetic regulation of these loci changes during the development of the liver and kidney, which control the small molecule composition of the blood, or the brain, whose physiology relies upon this process. Epigenetic regulation of these genes is also critical for understanding pharmacokinetics.

**Results:**

We profiled chromatin state at 1034 RSS/ADME genes in the mouse kidney, liver and brain at the embryonic and adult stages. Using the high-resolution chromatin mapping method CUT&RUN, we examined the activating histone modifications H3K4me3, H3K27ac and H3K9ac, and the repressive modification H3K27me3. Activating modifications were most dynamic at the chromatin level in the liver and least dynamic in the brain. Acetylated histone modifications were more dynamic overall than methylation marks in all three tissues. Hierarchical clustering demonstrated that a subset of RSS/ADME genes undergoes a coordinated program of activation during kidney and liver development that correlates with changes in transcript abundance.

**Conclusions:**

Defining the changes in chromatin that occur after birth within this gene set provides insight into tissue-specific regulation of RSS. Our findings carry implications for how the body acquires autonomous functionality through organ crosstalk mediated by transport of endogenous small molecules. Given their critical roles in ADME as well as handling of exogenous toxins, medications and metabolites derived from the gut microbiome, our analysis has ramifications for both precision pharmacology and diseases such as chronic kidney disease, metabolic syndrome and gout, in which dysregulation of RSS drives pathophysiology.

**Supplementary Information:**

The online version contains supplementary material available at 10.1186/s13072-025-00648-9.

## Background

Interorgan communication, a requirement for organismal autonomy, must be established between the late prenatal and postnatal periods [1–3]. This essential physiologic function relies upon the regulated exchange of thousands of blood-born organic small molecules. A network of ~ 1000 protein-coding genes mediates the process of “remote sensing and signaling” (RSS), largely by controlling transport of small molecules across cellular membranes [4, 5]. Hundreds of solute carrier (SLC) and ATP-binding cassette (ABC) transporter proteins, as well as many enzymes and transcription factors play central roles in organ crosstalk via small molecules [6, 7]. The RSS Theory emphasizes the importance of multi-specific, oligo-specific, and monospecific SLC and ABC transporters—as well as “drug“ metabolizing enzymes and their transcriptional regulators—in small molecule communication between organs [5, 8]. According to the RSS Theory, the RSS gene network largely overlaps with the related suite of genes that participate in the absorption, distribution, metabolism, and excretion (ADME) of small molecule pharmaceutical drugs [9, 10].

The small molecules under regulation by RSS include metabolites (e.g., TCA cycle intermediates), signaling molecules (e.g., cyclic GMP), nutrients, natural products (e.g., flavonoids), vitamins, and gut microbiome products [8, 11]. There is now considerable experimental data to support the concept that bile acids, gut microbiome metabolites, signaling lipids (e.g., short chain fatty acids, prostaglandins), uric acid and uremic toxins also participate in RSS and contribute to human disease when dysregulated [12–22].

Expression analyses have uncovered a large RSS network consisting of between 500 and 1000 proteins [4]. The genes that encode these proteins must be coordinately regulated during the transition from prenatal to postnatal development to coordinate this signaling axis between the liver, kidney and brain [23]. However, the contribution of changes in chromatin state, a major epigenetic mechanism for enacting tissue-specific, dynamic patterns of gene regulation during organ development, has yet to be directly examined in the context of genes that control RSS.

In this study, we employ the high-resolution, low background next-generation sequencing chromatin mapping method CUT&RUN (Cleavage Under Targets and Release Using Nuclease) [24] to examine activating and repressive modifications within RSS/ADME genes in the embryonic and adult mouse liver, kidney and brain. We find that the liver exhibits the greatest developmental dynamism in chromatin state, while the brain exhibits the least. We identify gene subsets that become epigenetically activated in a coordinated fashion within the kidney and liver. Our results represent the first attempt to investigate chromatin state across these genes, providing insight into the regulatory mechanisms that mediate organ crosstalk necessary for the establishment and maintenance of RSS.

## Methods

### Mice

Animals were cared for in accordance with NIH guidelines. All experimental methods were approved by the UCSD Institutional Committee on the Use and Care of Animals under the protocol number S20121. Mice on the Jackson Labs C57BL/6 background were used in all experiments. Animals were housed in a 12:12 light: dark cycle. For the adult timepoint, we used two male and two female biological replicates that were littermates. None of the main findings in this work varied by sex in the tissues we examined. For the embryonic timepoint, we combined several embryos, likely including both males and females for the 4 biological replicates.

### Nuclear isolation from kidney and liver

Adult mice (age 3 months) were deeply anesthetized with isofluorane before decapitation and extraction of the liver and kidney. Pregnant females that had been timed mated to produce embryonic mice of known age (embryonic day 16 in this case) were similarly processed. For the adult timepoint, the liver and kidney were further dissected before choosing the same lobe in the case of the liver and pole in the case of the kidney, inputting ~ 100 mg of tissue. Tissue was finely minced in lysis buffer (Nuclei EZ Lysis Buffer [Sigma-Aldrich CAT#N3408-200ML] supplemented with 1x Halt combined protease and phosphatase Inhibitor cocktail (ThermoFisher CAT#78430) and 10 mM sodium butyrate prior to mechanical homogenization with Dounce A pestle in 2 mL of Lysis Buffer. This suspension was incubated on ice for 5 min before centrifugation at 500 x g for 5 min, with acceleration and deceleration on the lowest setting, at 4 degrees. The supernatant was aspirated, and nuclei were resuspended in lysis buffer and incubated on ice for an additional 5 min prior to centrifugation. Examination of nuclei under DIC demonstrated abundant intact nuclei for all the tissues examined. Intact nuclei were nevertheless stained by trypan blue, confirming permeabilization. This process routinely yielded ~ 6 million nuclei from each adult sample after filtration. The embryonic tissues were carefully dissected from multiple embryos, inputting the entire organs for nuclear isolation.

### Nuclei isolation from embryonic brain

The whole brain of embryos from pregnant mice were dissected following deep isoflurane anesthesia and decapitation of mothers. Tissue (~ 20 mg) was minced in 1 mL HB + lysis buffer (0.25 M sucrose, 25 mM KCl, 5 mM MgCl₂, 20 mM Tricine-KOH, pH 7.8, supplemented with Halt protease inhibitor cocktail, 1 mM DTT, 10 mM sodium butyrate, 0.15 mM spermine, and 0.5 mM spermidine) and homogenized with 10 strokes using the pre-chilled 7 mL Dounce A homogenizer. IGEPAL CA-630 was added to a final concentration of 0.3%, followed by 10 additional strokes. Homogenates were incubated on ice for 5 min and centrifuged at 500 × g for 5 min at 4 °C with the lowest acceleration and deceleration settings. Pelleted nuclei were resuspended in 1 mL wash buffer (20 mM HEPES-KOH, pH 7.5, 150 mM NaCl, 0.15 mM spermidine and Halt protease inhibitors), washed by a second centrifugation, and filtered through a 40 μm mesh. Nuclei were quantified using a Countess automated counter.

### Nuclei isolation from adult brain

Adult mice (age 3 months) were deeply anesthetized with isoflurane prior to decapitation and dissection of the forebrain. Tissue (~ 50 mg) was finely minced in 1 mL of HB + buffer (0.25 M sucrose, 25 mM KCl, 5 mM MgCl₂, 20 mM Tricine-KOH, pH 7.8, supplemented with 1x Halt protease inhibitor cocktail, 1 mM DTT, 0.15 mM spermine, and 0.5 mM spermidine) on ice. Minced tissue and an additional 2.76 mL HB + were transferred to a 7 mL pre-chilled Dounce homogenizer and subjected to 10 strokes using the A pestle. IGEPAL CA-630 was added to a final concentration of 0.3%, followed by 10 additional strokes with the A pestle. The homogenate was incubated on ice for 5 min and gently filtered through a 40 μm mesh by gravity flow. Then the homogenate was mixed with 1.7 mL of 50% iodixanol solution to achieve a final iodixanol concentration of ~ 15%. Subsequently, 2 mL of 25% iodixanol was gently underlain beneath the tissue homogenate to form two density layers. Samples were centrifuged at 4000 × g for 30 min at 4 °C (acceleration set to 6 out of 9, deceleration at the lowest setting). The upper aqueous phase was carefully removed and pelleted nuclei were gently collected. Isolated nuclei were resuspended in 1 mL of wash buffer (20 mM HEPES-KOH, pH 7.5, 150 mM NaCl, 0.5 mM spermidine and 1x Halt protease inhibitor) and filtered through a 40 μm mesh. Nuclei were quantified using a Countess automated cell counter.

### Native CUT&RUN

For CUT&RUN performed under native conditions, nuclei were resuspended in wash buffer (20 mM HEPES pH 7.5, 150 mM NaCl, 0.5 mM spermidine supplemented with Roche complete EDTA-free protease inhibitor tablet CAT#11873580001). Pelleted nuclei were resuspended twice in wash buffer and subjected to centrifugation at 500 x g for 5 min (lowest acceleration and deceleration parameters). Nuclei were resuspended in 1 mL wash buffer, filtered by gravity through a 40 μm mesh, and counted with a Countess automated cell counter (Invitrogen). In general, 500,000 nuclei were used in each CUT&RUN experiment, and the cellular input was kept the same across all replicates. Nuclei were bound to Concanavalin A-coated magnetic beads (CUTANA CAT# 21-1401). Following bead binding, the supernatant was discarded, and the bead-nuclei slurry was resuspended in 75 µL of antibody buffer (wash buffer supplemented with 0.005% digitonin and 2 mM EDTA) containing primary antibodies at a concentration of 1:75. Antibodies used in this study were H3K4me3 (CST CAT# 9751 S, RRID: AB_2616028), H3K27ac (Active Motif CAT# 39034), H3K9ac (Active Motif CAT# 39138, RRID: AB_2561017) and H3K27me3 (CST CAT# 9733 S RRID: AB_2616029). Antibody incubation was carried out overnight at 4 °C on a nutator. Samples were washed with cold cell permeabilization buffer (Wash buffer with 0.005% digitonin) to remove unbound antibody and then incubated with 1:50 Protein A/G-MNase (Epicypher CAT# 15-1016) in cell permeabilization buffer for 15 min at room temperature on a shaker. Samples were washed with cold cell permeabilization buffer and then resuspended in 50 µL cell permeabilization buffer containing 2 mM CaCl_2_. Samples were digested for 2 h at 4 °C on a nutator. The digestion reactions were quenched with 33 µL of STOP buffer (340 mM NaCl, 20 mM EDTA pH 8, 4 mM EGTA pH 7.7, RNase A 0.05 mg/mL, Glycogen 0.05 mg/mL). Samples were then incubated for 10 min at 37 °C to release digested chromatin fragments. The supernatant containing CUT&RUN fragments was transferred to fresh tubes and DNA was purified via column purification.

### CUT&RUN library Preparation and sequencing

Library preparation for CUT&RUN experiments was performed according to the manufacturer’s specifications (New England Biolabs, CAT# E7645L) using SPRIselect beads. The concentration of DNA yielded from CUT&RUN was measured on a Qubit device and between 15 and 30 ng of input DNA was used for library preparation, which was performed at half scale, with the amount of input DNA kept constant for all samples using the same antibody. 14 PCR cycles were used in the final amplification with Illumina-compatible adaptors, universal i5 primer and barcoded i7 primers. After measuring the concentration of the amplified product on Nanodrop, all samples were diluted to the same concentration for a given antibody, ranging from 10 ng/µL to 40 ng/µL. Tapestation was performed to determine the precise concentration and fragment distribution range and to ensure there were not significant adaptor dimers. Samples were pooled for sequencing 100 bp reads in paired-end configuration on an Illumina NovaSeqX platform at the UCSD Institute for Genomic Medicine. We aimed for > 30 million reads for all samples except for H3K4me3, for which 15 million reads were adequate given the narrow distribution of this histone modification.

### CUT&RUN data processing

We used a modified version of CUT&RUNtools [25, 26]. Compressed sequenced reads in fastq.gz format were first trimmed using Trimmomatic v.0.39 [27]. Samples were then aligned to the mm10 genome using Bowtie2 v.2.3.4.1 [28]. BAM files were then sorted and unmapped fragments removed using SAMtools v.1.14 [29]. Alignment files were then converted to .bigwig files using the bamCoverage tool in Deeptools v.3.5.0 [30] with 50 bp bins. Peak calling was performed using SEACR (Sparse Enrichment Analysis for CUT&RUN) v.1.1 [31]. Bigwig and .bed files were examined and visualized in Integrated Genome Viewer [32, 33]. Regional overlap between different samples was obtained using the ‘intersect’ functionality of BEDTools v.2.29.2 [34].

### Normalization of CUT&RUN data

This method was previously described [35]. Briefly, after parsing the genome into 50 bp bins, we identified local maxima and generated a blacklist of 272 massive intergenic peaks representing spurious alignment artifacts. The height of the leftover authentic peaks was quantified and the value of the height for the 99th percentile peak recorded. The ratio of the values of the 99th percentile peaks between samples served as a scaling factor by which the height of every bin in one sample could be multiplied to normalize samples to one another.

### Genome-Wide aggregate heatmaps

Genome-wide heatmaps were generated using Deeptools v.3.5.0. Plots centered around peak summits were produced in reference to .bed files output from peak calling analysis of CUT&RUN data from mouse tissue. computeMatrix was used to represent each locus from bigwig CUT&RUN data, where the rows were loci and the columns were 50 bp bins. Matrices were subsequently plotted as heatmaps using the plotHeatmap tool. For metagene plots, in which the signal in bigwig tracks was scaled to gene length, the metagene flag was used during computeMatrix. plotHeatmap was then used to visualize the abundance of different histone PTMs.

### Quantitation of CUT&RUN signal

Quantitation was performed in a gene-centric manner. For each histone mark within a single tissue, peak regions from both embryonic (E16) and adult (3 month) stages identified by SEACR were merged to define the genomic intervals to include for each of the 1034 RSS/ADME genes. Boundaries for each gene were defined as the gene body plus 5 kb upstream of the transcription start site (TSS) and 2 kb downstream of the transcription end site (TES). To generate a single measurement per gene and histone modification, a length-weighted average was computed across all intervals assigned to that gene. Normalized bigwig tracks were then quantitated over these RSS/ADME-associated regions of epigenomic activity at either timepoint to enable direct comparisons across loci of varying size and complexity. Quantitation was performed across four biological replicates per stage, yielding gene-level signal values for each histone PTM at embryonic and adult timepoints. These values in Table S1 were subsequently used for scatterplots, volcano plots and principal component analysis (PCA).

### Hierarchical clustering

Gene-level signal values obtained as described above were used for differential analysis with limma voom [36] and edgeR [37]. For each histone PTM–tissue combination, genes were assigned to bins according to the direction of fold change and statistical significance (α = 0.05): +1 for significantly increased, − 1 for significantly decreased, and 0 for non-significant. The resulting gene by modification score matrix was subjected to agglomerative hierarchical clustering (Ward’s linkage, Euclidean distance). For each tissue (kidney, liver, brain), the dendrogram was cut into five clusters; combined clustering across all three tissues was cut into six clusters. Differential analysis was performed in R with limma voom, and clustering and visualization were carried out in Python 3 using pandas, scipy, and matplotlib.

### Comparison of RSS/ADME genes to non-RSS/ADME genes

Gene counts for each gene set (1034 RSS/ADME genes versus 18603 non-RSS/ADME genes) were categorized as ‘significantly increased’, ‘significantly decreased’, or ‘non-significant/unchanged’ based on the limma voom analysis described above. We then performed chi-square testing on the 2 × 3 contingency table using the scipy.stats library in Python3.

### RNAseq

We used the ENCODE consortium [38, 39] as a source for transcriptomic data. All data were the product of RNAseq analysis on polyA selected mRNA. For the embryonic day 16 timepoint, specific sources of gene counts were the following datasets: kidney (ENCFF264KWG), liver (ENCFF620ZGA) and brain (ENCFF029UVS). For the adult timepoint, specific sources of gene counts were the following datasets: kidney (ENCFF240PJE), liver (ENCFF680TRV) and brain (ENCFF468CXD). Transcripts per million (TPM) values were sorted according to the clusters defined for histone modifications and presented at the log_2_ of the ratio of adult/embryonic values for each RSS/ADME gene.

### Correlation network construction and analysis

For each tissue (kidney, liver, brain), we began with the matrix of developmental log fold changes (age 3 month / age E16) for 1034 RSS/ADME genes across four histone marks (H3K4me3, H3K27ac, H3K9ac, H3K27me3). Pairwise Spearman rank correlations were computed between every gene’s four-dimensional fold change vector. An undirected, weighted network was then assembled by retaining edges with correlation coefficient ≥ 0.9, assigning each edge a weight equal to its Spearman ρ. Network density was calculated as the number of observed edges divided by the total possible.

### Hub identification and comparison

Node strength (the sum of absolute Spearman ρ weights per gene) was calculated within each tissue’s weighted correlation network. To define a data driven hub threshold, we pooled all node strengths across kidney, liver and brain and examined their distribution, identifying the 80th percentile value (strength ≥ 166.08) as the cutoff for significant hubs. To quantify shared regulatory architecture, pairwise Jaccard similarity coefficients were then computed between each pair of tissue-specific hub sets.

### Graphing

All plots were generated in GraphPad Prism 10 and modified in Adobe Illustrator 2021.

## Results

### Selection of RSS/ADME gene set

**Fig. 1:**
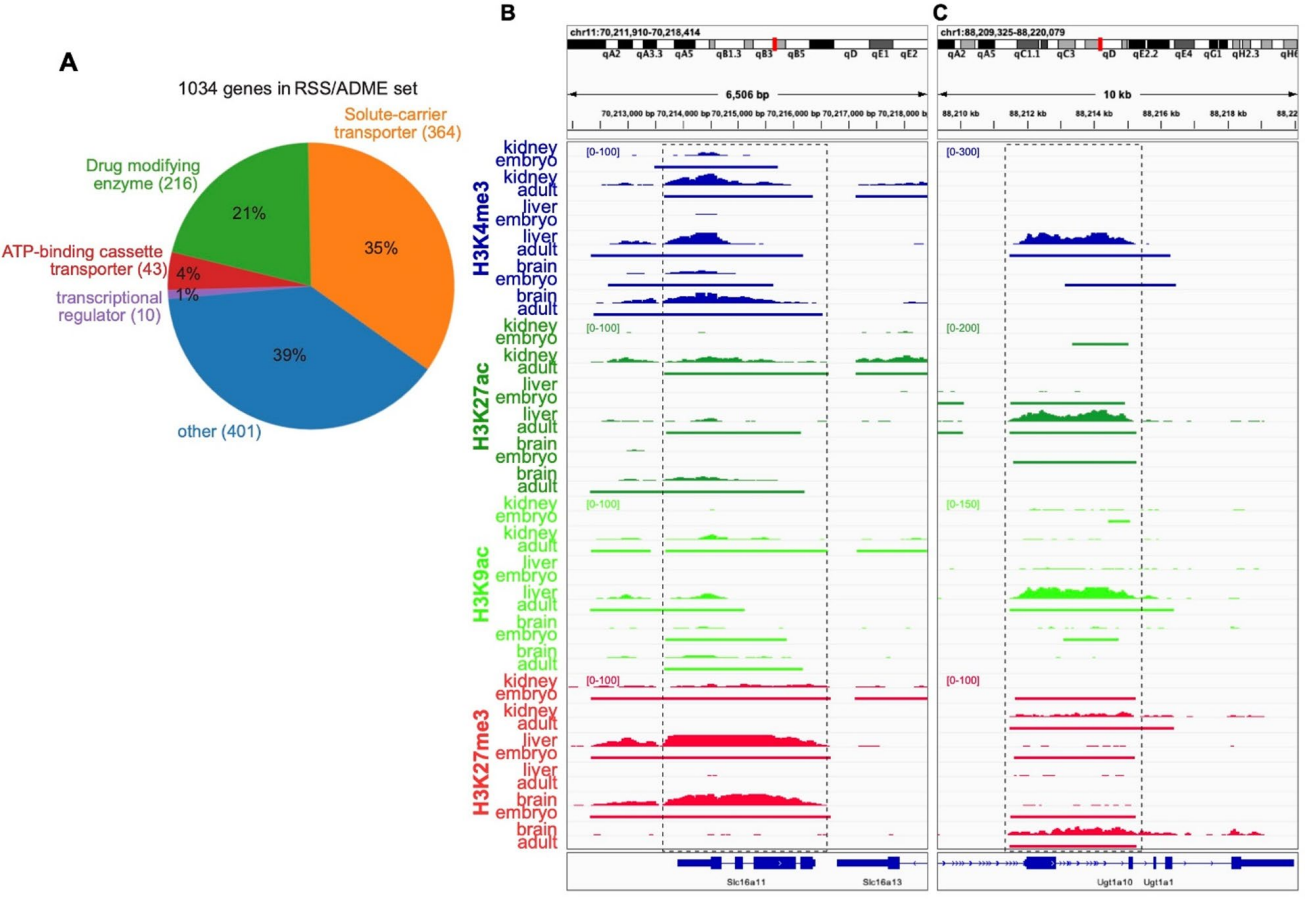
Tissue-general and tissue-specific developmental remodeling of chromatin state at RSS/ADME genes. **A** The dataset was curated based on previous representations of the Remote Sensing and Signaling (RSS) Network [4] and available lists of proteins involved in pharmacokinetics and/or absorption, distribution, metabolism, excretion (ADME) [40–42]. Certain transcriptional regulators were also added based on literature studies [43, 44]. The “other” category includes enzymes and proteins hypothesized but not proven to be linked to the RSS Network or ADME pathways [40, 42]. **B** CUT&RUN tracks of H3K4me3, H3K27ac, H3K9ac, and H3K27me3 data at the *Slc16a11* locus in kidney, liver and brain at the embryonic (embryonic day 16, E16) and adult stages (age 3 months). Below each bigwig track is a BED file track showing peak regions called by the SEACR algorithm [31]. **C** *Ugt1a* gene cluster

In order to examine epigenetic regulation of genes required for RSS, we began by defining our list of 1034 genes (Table S1). This list was constructed based upon a previously published RSS network [4], established ADME gene lists [4, 40–42], and additional transcriptional regulators in the literature that are associated with the regulation of the aforementioned genes [43, 44]. The resulting classification includes transporters, drug-metabolizing enzymes (DMEs), and transcriptional regulators, with an additional “other” category for proteins likely to be linked to RSS list but not completely validated [40, 42]. The proportional representation of these different classes of loci is summarized in Fig. 1A.

### Tissue-general and tissue-specific developmental changes in key regulatory histone modifications

To examine regulatory histone modifications at RSS/ADME loci, we employed the high-resolution chromatin mapping method CUT&RUN (Cleavage Under Targets and Release Using Nuclease) [24, 45]. We profiled the liver, kidney and brain at two timepoints – embryonic day 16 (E16, embryonic) and age 3 months (adult), including four biological replicates at each timepoint. These stages bookend the period that encompasses post-organogenesis tissue specification, post-partum onset of independent metabolism, and ultimately the arrival at the mature state of organ functionality including interorgan communication [1, 46]. E16 was selected as the embryonic timepoint because it coincides with the completion of a relatively stable stage (in terms of gene expression) that occurs from E13-16 and corresponds to the period of tubule formation during kidney development [47].

After isolating nuclei from these tissues, we applied antibodies to detect four post-translationally modified histone proteins that confer key gene regulatory functions: H3K4me3 (histone 3 trimethylated at Lys4, typically enriched at promoters of actively transcribed genes); H3K9ac (histone 3 acetylated at Lys9, typically enriched at active promoters and enhancers); H3K27ac (histone 3 acetylated at Lys27, typically enriched at active promoters and enhancers); and H3K27me3 (histone 3 trimethylated at Lys27, typically found in gene promoters and/or gene bodies which are silenced in facultative heterochromatin). The presence or absence of these modifications in combination with one another contributes to the regulatory state of individual protein-coding loci. We processed reads through a modified version of the established Cut&RunTools pipeline [25, 26], normalizing data across replicates and timepoints for a given histone modification in each organ, using a previously described percentile-based algorithm [35]. For calling peak regions, we used the SEACR (Sparse Enrichment Analysis for CUT&RUN) [31] algorithm.

Comparing the abundance and distribution of these modifications at the gene *Slc16a11* showed that these regulatory histone modifications were remodeled within both the promoter region and across the gene body during the development of these tissues (Fig. 1B). *Slc16a11* exhibited a coordinated program of activation across the three tissues that included deposition of the activating modifications H3K4me3, H3K27ac and H3K9ac accompanied by loss of the repressive modification H3K27me3, although the latter effect was less pronounced in the kidney. In contrast, genes like *Ugt1a1* (encoding the metabolic enzyme UDP glucuronosyltransferase) underwent developmental deposition of activating modifications specifically in the liver (Fig. 1C). In contrast, the brain and kidney experienced deposition of repressive H3K27me3 that did not occur in the liver. These examples led us to explore how the regulation of chromatin state might differ between the kidney, liver and brain across the entire set of 1034 RSS/ADME genes.

### Within RSS/ADME genes, methylated histones are bidirectionally dynamic to the greatest degree in the liver

To gain a more quantitative understanding of how the activating modification H3K4me3 and the repressive modification H3K27me3 are regulated across RSS/ADME loci, we performed a series of aggregate analyses. After calling peaks in CUT&RUN data using SEACR, we used BEDtools [34] to combine peak regions identified in any of the four replicates at either timepoint for a given tissue and histone modification. We then associated these peak regions with each RSS/ADME gene, including regions residing within 5 kb upstream of the transcription start site (TSS) or 2 kb downstream of the transcription end site (TES). In this way we included the regulatory regions where these modifications are concentrated. Examining just peak regions avoided a potential confounding factor when comparing genes of different length in which the signal originating from regulatory regions (which tend to be of similar size) could be diluted across an entire coding regions (which vary widely in length). The measured abundance of each histone modification was then scaled according to the combined length of all peak regions linked to a given gene (Table S2).

**Fig. 2:**
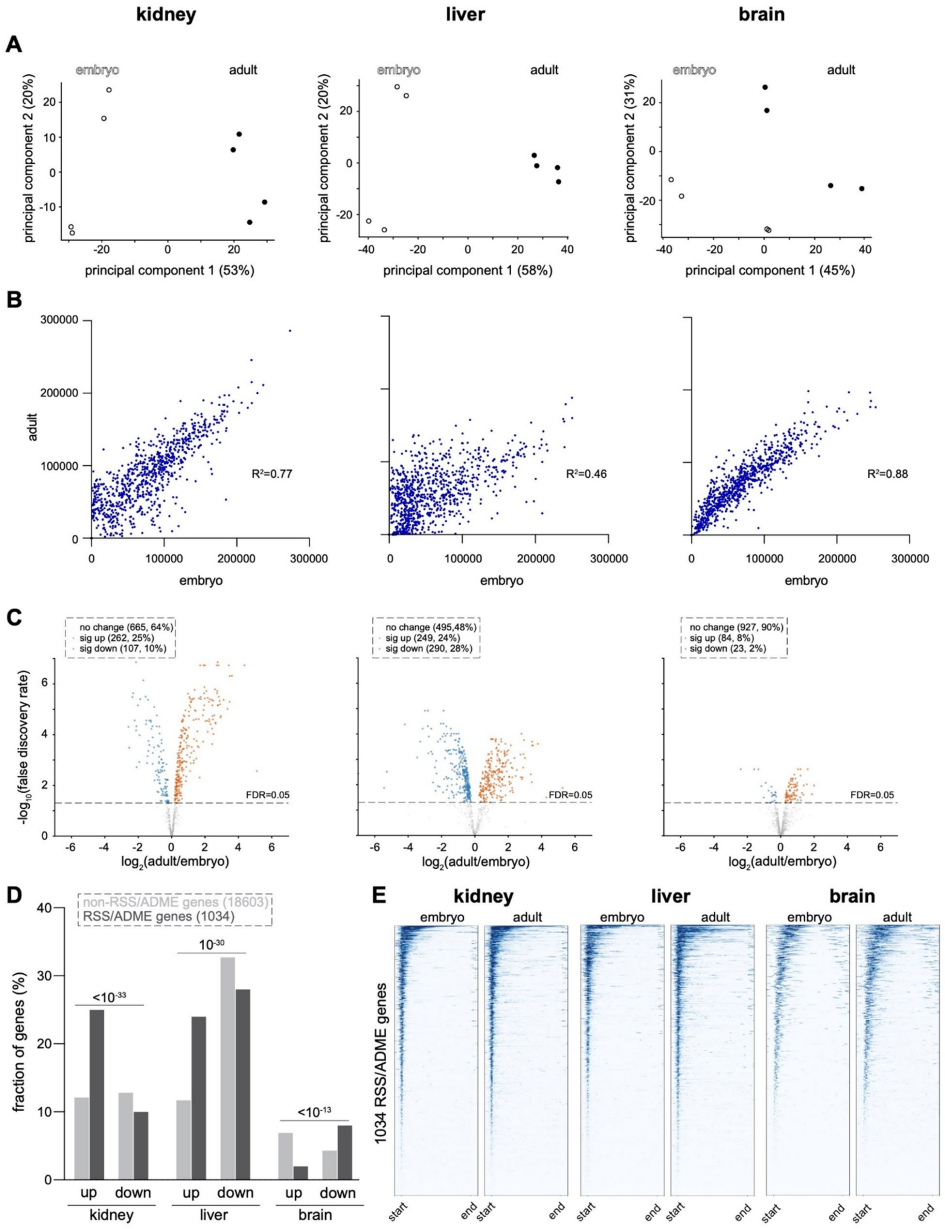
Promoter-associated H3K4me3 is most dynamic in liver. **A** Principal component analysis (PCA) of quantitated H3K4me3 CUT&RUN signal in normalized biological replicates of each organ at the embryonic and adult stages. **B** Scatterplots of H3K4me3 CUT&RUN signal comparing the embryonic and adult stages. Each dataset reflects the average of 4 biological replicates. For details of the quantitative methodology, please refer to the Methods section. **C** Differential analysis of replicate CUT&RUN data at the embryonic and adult timepoints using limma-voom [36] and EdgeR [37]. **D** Comparison of the fraction of RSS/ADME versus non-RSS/ADME genes with significantly increased (up) or decreased (down) H3K4me3 at the adult versus embryonic stages. P-values by Chi-square test. **E** Metagene aggregate heatmap plots of H3K4me3 CUT&RUN data in RSS/ADME loci at embryonic and adult timepoints in each organ

After computing the abundance of H3K4me3 within 1034 RSS/ADME genes, we performed principal component analysis (PCA) on the resulting data. PCA grouped experimental samples from the non-neural organs by timepoint along the first component, which accounted for most of their variation (Fig. 2A). Clusters separated to a lesser degree in the brain, suggesting greater similarity in H3K4me3 between the two timepoints. Scatterplots comparing the abundance of H3K4me3 in each organ at the two timepoints demonstrated that the values were more similar within the kidney and brain, resulting in comparatively high R^2^ values of 0.77 and 0.88, respectively (Fig. 2B). In contrast, scatterplots depicting liver data were much more dispersed, resulting in a lower R^2^ value of 0.46. These observations indicate that H3K4me3 is established early and subsequently maintained in the kidney and brain, whereas the degree of dynamism is higher in the liver, with some loci gaining and others losing H3K4me3.

We next used the limma package [36] to statistically compare the abundance of H3K4me3 at the early versus late timepoints (Table S3). Volcano plots show H3K4me3 abundance across RSS/ADME genes in relation to statistical significance (Fig. 2C). Setting a false-discovery rate threshold of < 0.05, we found that many more RSS/ADME genes underwent a significant increase or decrease in H3K4me3 in the liver than in the kidney, with the brain having the fewest loci that exhibited a significant degree of developmental dynamism. Interestingly, in the liver the number of loci that underwent an increase in H3K4me3 was approximately equal to the number of loci that underwent a decline, whereas in the kidney the balance was shifted toward increasing abundance of H3K4me3. To determine if these changes were specific to the RSS/ADME gene set, as opposed to a general feature of chromatin regulation development of these tissues, we also analyzed the 18,603 non-RSS/ADME protein coding genes using the same data as input and the same quantitative approach as for the RSS/ADME gene set. In the kidney and liver, RSS/ADME genes were approximately twice as likely to undergo a significant increase in the abundance of H3K4me3 in comparison to non-RSS/ADME genes, a difference that was not observed in the brain (Fig. 2D). These data suggest that RSS/ADME genes undergo a specific program of developmental activation at the level of chromatin modifications.

As an alternative method of visualizing H3K4me3 CUT&RUN data across the collection of RSS/ADME genes, we generated aggregate heatmaps depicting the distribution of H3K4me3 across all 1034 RSS/ADME loci in metagene configuration, which standardizes the signal to a constant gene length for each locus (Fig. 2E). We included 2 kb upstream and downstream of the TSS and TES to incorporate regulatory regions. As expected, the H3K4me3 signal was centered near the TSS in every tissue, although the signal was noticeably shifted downstream in the brain, potentially reflecting alternative start codon usage.

**Fig. 3:**
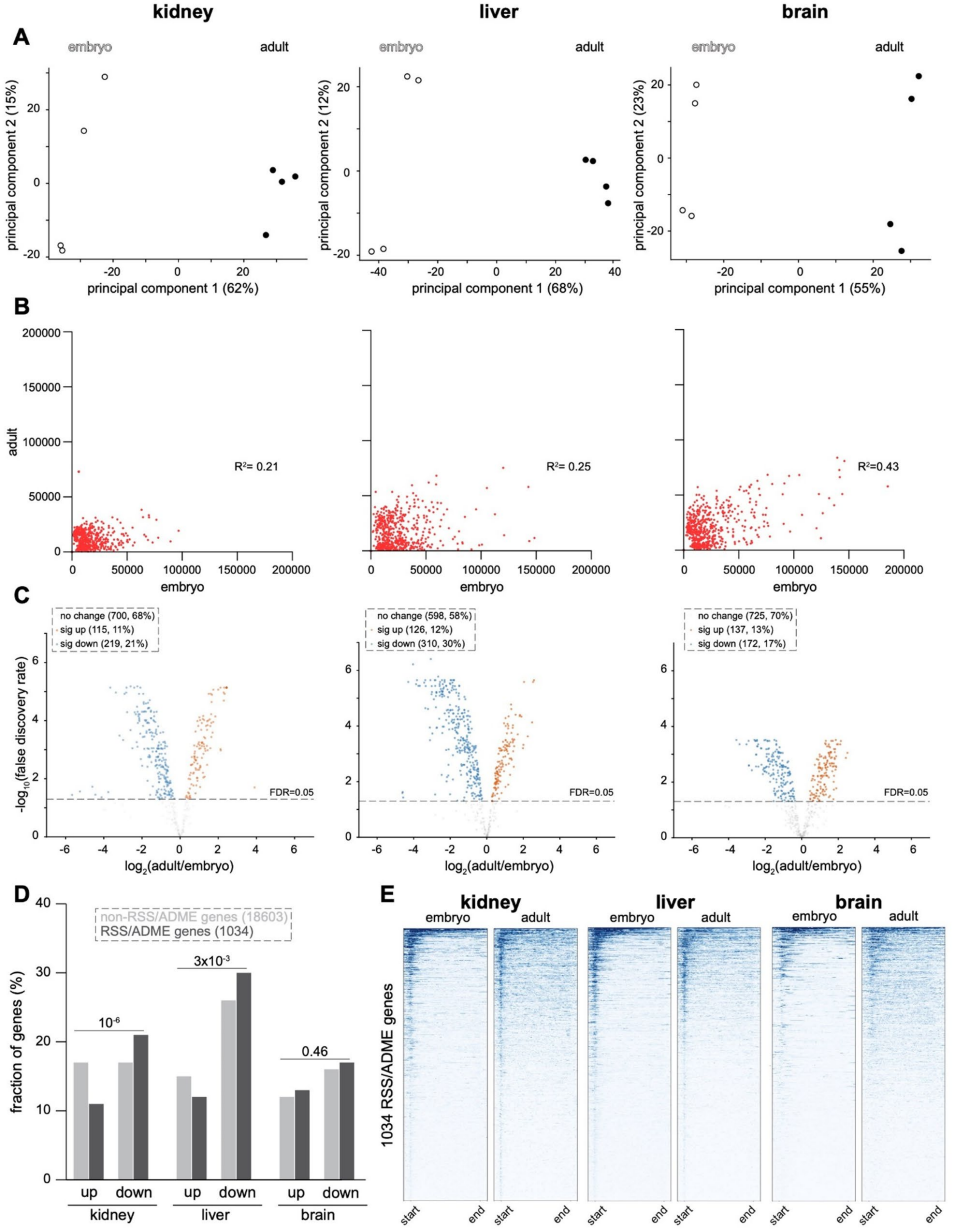
H3K27me3 is redistributed during development from promoters to gene bodies and is most dynamic in liver. **A** Principal component analysis (PCA) of quantitated H3K27me3 CUT&RUN signal in normalized biological replicates of each organ at the embryonic and adult stages. **B** Scatterplots of H3K27me3 CUT&RUN data comparing the embryonic and adult stages. Each dataset reflects the average of 4 biological replicates. **C** Differential analysis of replicate CUT&RUN data at the embryonic and adult timepoints using limma-voom and EdgeR. **D** Comparison of the fraction of RSS/ADME versus non-RSS/ADME genes with significantly increased (up) or decreased (down) H3K27me3 at the adult versus embryonic stages. P-values by Chi-square test. **E** Metagene aggregate heatmap plots of H3K27me3 CUT&RUN data in RSS/ADME loci at embryonic and adult timepoints in each organ

We next examined the degree to which the modification H3K27me3, a key mediator of gene repression in facultative heterochromatin that is deposited by the polycomb-repressive complex 2, [48] changes across RSS/ADME loci during the development of each tissue. PCA clustered replicates by timepoint in all three tissues along component 1 (Fig. 3A). Plots comparing the abundance of H3K27me3 between the embryonic and adult timepoints demonstrated greater dynamism in the liver and kidney (reduced R^2^ values) than in the brain (Fig. 3B). In comparison to H3K4me3, R^2^ values were lower for H3K27me3, indicating that H3K27me3 is overall more dynamic in all three organs (Fig. 3B, Table S2). Volcano plots demonstrated that a greater fraction of RSS/ADME loci experienced a significant change in H3K27me3 during the development of the liver, followed by the kidney and brain (Fig. 3C, Table S3). Interestingly, in each tissue there were more genes for which H3K27me3 declined significantly than for which this modification increased, indicating that maturation is associated with derepression of RSS/ADME genes in general. Compared to non-RSS/ADME genes, members of the RSS/ADME gene set were somewhat less likely to gain H3K27me3 and somewhat more likely to lose H3K27me3 in the kidney and liver (Fig. 3D), a finding that is compatible with a developmental program of activation.

Aggregate heatmaps showed a qualitative change in the distribution of H3K27me3, in which this modification transitioned from being promoter-associated at the embryonic stage to become more evenly distributed across the coding region of labeled genes at the adult stage (Fig. 3E). This pattern occurred in each of the examined tissues, indicating this transformation is a fundamental aspect of facultative heterochromatin formation, which is thought to form by a process of initial nucleation followed by spreading to adjacent regions [49, 50].

Taken together, our analysis of histone methylation marks demonstrates that both the activating H3K4me3 and the repressive H3K27me3 modifications are more developmentally dynamic among RSS/ADME genes in the liver and kidney than in the brain.

### Acetylation of RSS/ADME genes is overall more dynamic than histone methylation, especially in the liver

**Fig. 4:**
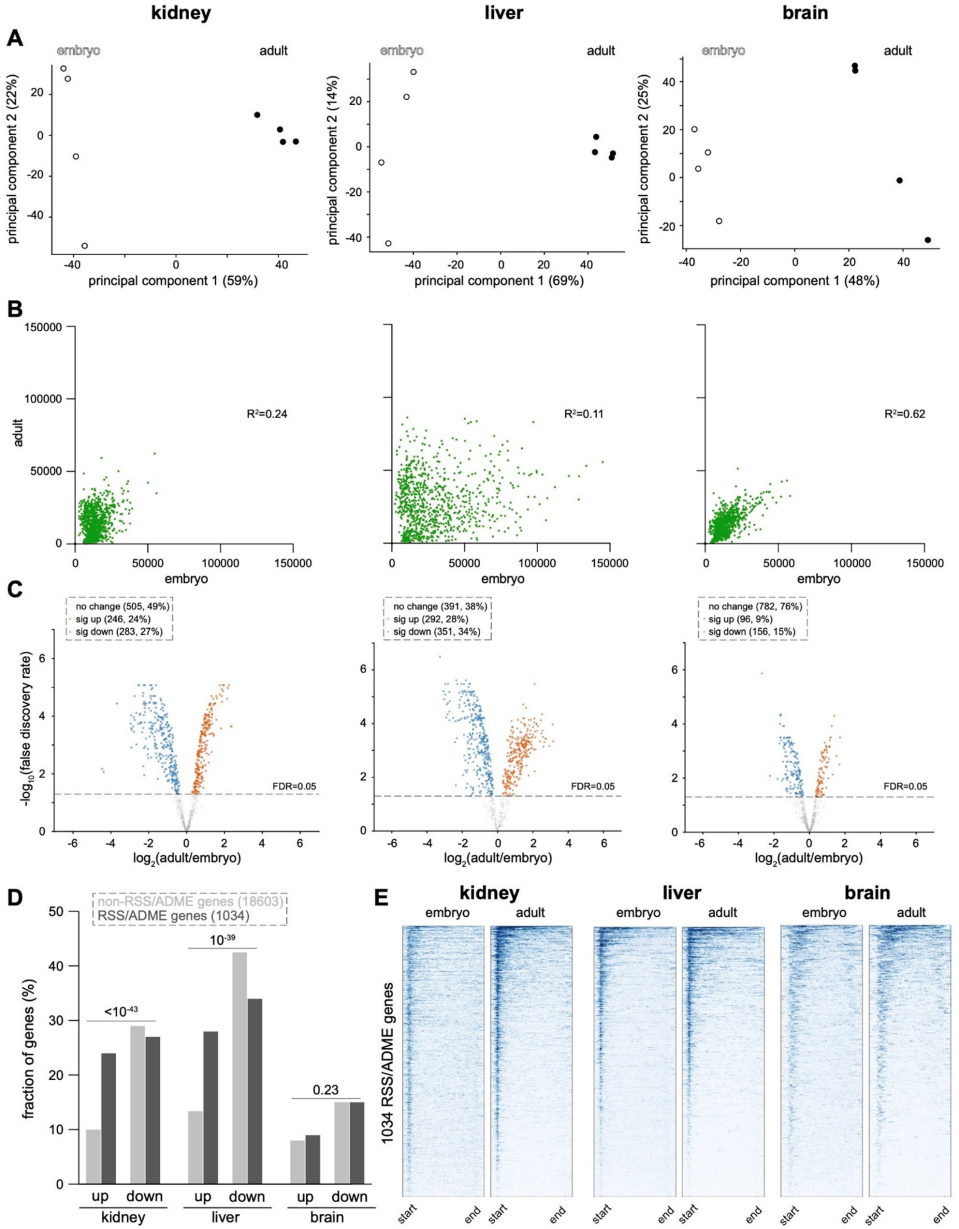
H3K27ac undergoes tissue-specific redistribution with promoter-associated enrichment in the kidney. **A** Principal component analysis (PCA) of quantitated H3K27ac CUT&RUN signal in normalized biological replicates of each organ at the embryonic and adult stages. **B** Scatterplots of H3K27ac CUT&RUN signal comparing the embryonic and adult stages. Each dataset reflects the average of 4 biological replicates. **C** Differential analysis of replicate CUT&RUN data at the embryonic and adult timepoints using limma-voom and EdgeR. **D** Comparison of the fraction of RSS/ADME versus non-RSS/ADME genes with significantly increased (up) or decreased (down) H3K27ac at the adult versus embryonic stages. P-values by Chi-square test. **E** Metagene aggregate heatmap plots of H3K27ac CUT&RUN data in RSS/ADME loci at embryonic and adult timepoints in each organ

Histone acetylation is one of the hallmarks of euchromatin, as this post-translational modification tends to increase the accessibility of chromatin to the transcriptional machinery to promote gene expression [51]. For instance, the presence of promoter- and enhancer-associated H3K27ac drives transcription of nearby genes [52]. Accordingly, H3K27ac is one of the key epigenetic modifications capable of reprogramming gene expression states during tissue development [53]. Differences in the patterns of H3K27ac also contribute to the adoption of gene expression profiles that underlie cell- and tissue-specific transcriptomes. After mapping and quantitating H3K27ac by CUT&RUN in the kidney, liver and brain, PCA segregated replicate data by timepoint along the first principal component in all tissues (Fig. 4A). Scatterplots of H3K27ac abundance within RSS/ADME genes demonstrated that the R^2^ value for each tissue was much lower than for the methylated histone modifications, giving rise to comparatively dispersed scatterplots (Fig. 4B, Table S2). Dynamism was greatest in the liver (R^2^ of 0.11), followed closely by the kidney (R^2^ of 0.24), with conspicuously less change in the acetylation state of RSS/ADME genes in the brain (R^2^ of 0.62). Volcano plots also demonstrated the greatest degree of bidirectional dynamism in the liver, with the majority of RSS/ADME undergoing either significant increases or decreases in H3K27ac (Fig. 4C, Table S3). A smaller majority of RSS/ADME genes in the kidney exhibited a significant change in the abundance of H3K27ac, with many fewer loci changing in the brain. Comparing RSS/ADME genes to non-RSS/ADME genes demonstrated that, in the kidney and liver, RSS/ADME genes were about twice as likely to experience a significant increase in the abundance of H3K27ac, an effect which was not observed in the brain (Fig. 4D). These data are consistent with a model in which RSS/ADME genes undergo a specific program of developmental activation at the chromatin level. Interestingly, in aggregate heatmaps, the pattern by which H3K27ac became reorganized was qualitatively different in the kidney, as this modification went from being diffusely distributed throughout gene bodies to become consolidated within promoter regions (Fig. 4E).

**Fig. 5:**
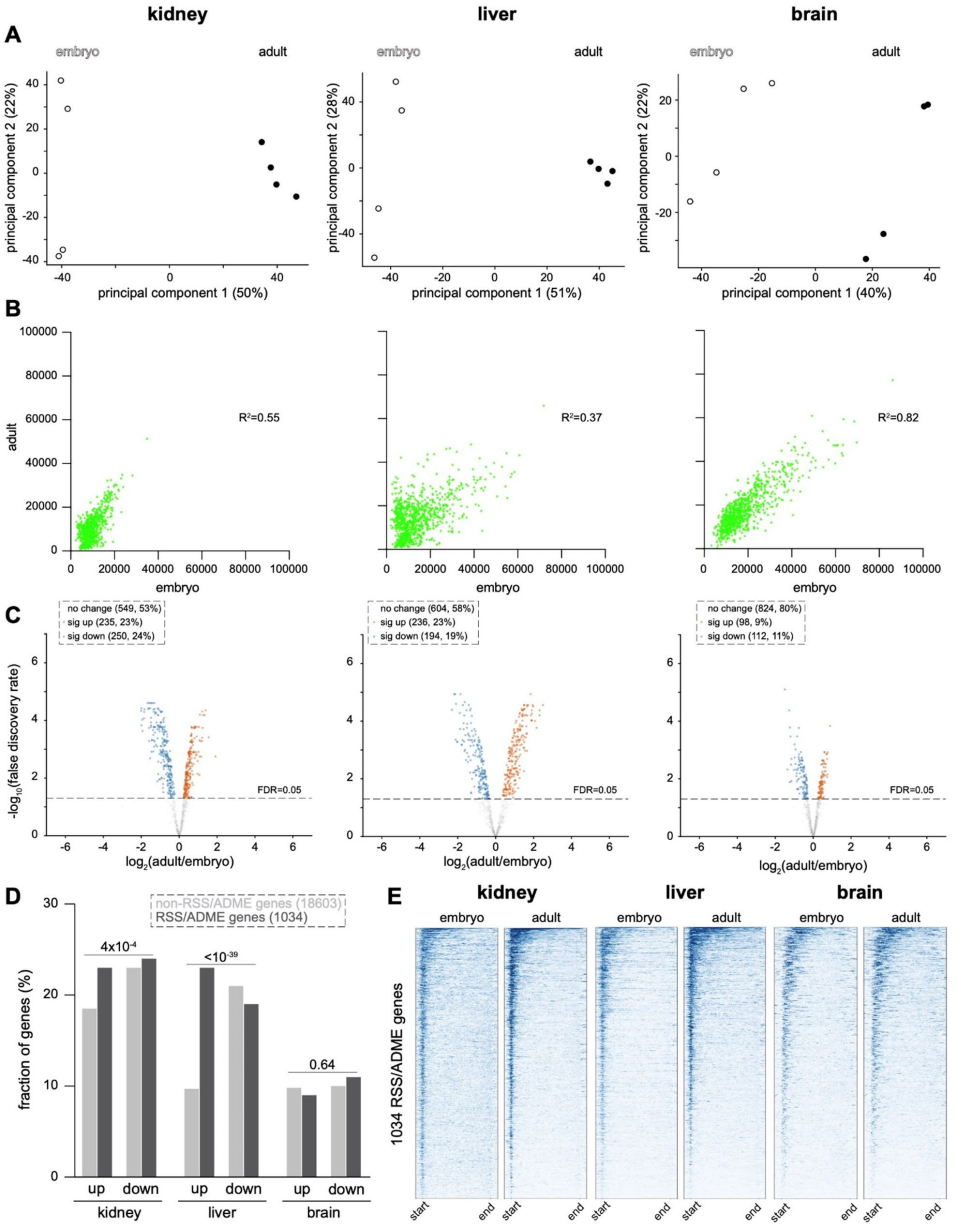
H3K9ac is more dynamic in the kidney and liver than the brain. **A** Principal component analysis (PCA) of quantitated H3K9ac CUT&RUN signal in normalized biological replicates of each organ at the embryonic and adult stages. **B** Scatterplots of H3K9ac CUT&RUN signal comparing the embryonic and adult stages. Each dataset reflects the average of 4 biological replicates. **C** Differential analysis of replicate CUT&RUN data at the embryonic and adult timepoints using limma-voom and EdgeR. **D** Comparison of the fraction of RSS/ADME versus non-RSS/ADME genes with significantly increased (up) or decreased (down) H3K9ac at the adult versus embryonic stages. P-values by Chi-square test. **E** Metagene aggregate heatmap plots of H3K9ac CUT&RUN data in RSS/ADME loci at embryonic and adult timepoints in each organ

We also examined the acetylated histone H3K9ac, which is associated primarily with the promoter regions of actively transcribed genes [54]. Similar to the other histone modifications, PCA analysis of H3K9ac data demonstrated clustering by timepoint along principal component 1 (Fig. 5A). Scatterplots depicting H3K9ac abundance showed greatest dynamism in the liver (R^2^ = 0.37) and least dynamism in the brain (R^2^ = 0.82) (Fig. 5B, Table S2). Likewise, volcano plots from the brain uncovered the fewest genes with significant changes during development (Fig. 5C, Table S3). Comparing RSS/ADME genes to non-RSS/ADME genes demonstrated that, in the non-neural organs, RSS/ADME genes were more likely to undergo a significant increase in the abundance of H3K9ac, an effect which was particularly pronounced in the liver and not observed in the brain (Fig. 5D). Similar to H3K27ac, in the kidney H3K9ac underwent a process of refinement in which H3K9ac was initially distributed throughout gene bodies before becoming enriched within promoter regions (Fig. 5E).

Collectively, these observations demonstrate that histone acetylation is generally more dynamic than histone methylation in RSS/ADME genes across all three tissues. Furthermore, these acetylation marks changed to the greatest degree during the development of the liver followed by the kidney. In the brain, RSS/ADME genes were comparatively less dynamic in their chromatin state relative to the non-neural organs, which are more directly integrated into the physiologic process of interorgan communication [1].

### Hierarchical clustering reveals distinct patterns of chromatin reorganization at RSS/ADME genes in different tissues

**Fig. 6:**
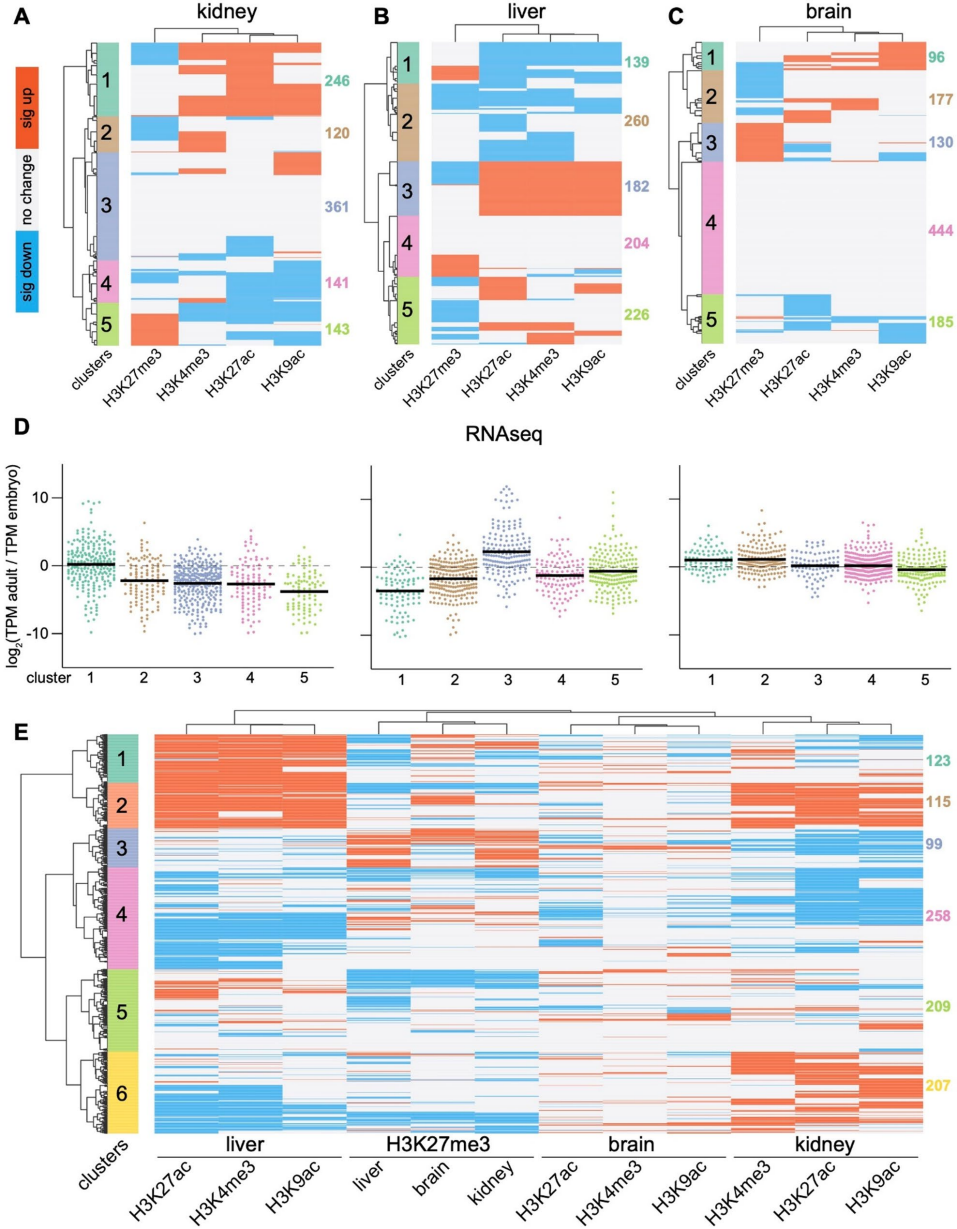
Hierarchical clustering of CUT&RUN data identifies a large subset of RSS/ADME genes that becomes activated during kidney and liver development. **A** Unsupervised hierarchical clustering of developmentally differential CUT&RUN signal for H3K4me3, H3K27ac, H3K9ac and H3K27me3 at SEACR-defined peak regions assigned to RSS/ADME loci in kidney. Gene-level signals were analyzed using limma-voom and binned as + 1 (significant increase), -1 (significant decrease), or 0 (no change). Five clusters are indicated and color coded. **B** Clustering of liver data. **C** Clustering of brain data. **D** Transcriptomic assessment of chromatin-based clustering. For each cluster, the transcripts per million (TPM) of each included gene is depicted as the log_2_ of the ratio of the mRNA abundance at the adult/embryonic timepoints. **E** Combined clustering of kidney, liver and brain for all histone modifications, followed by partitioning into six clusters to capture both shared and tissue-restricted patterns of chromatin dynamism

To determine how activating and repressive histone modifications at RSS/ADME genes might be coordinately regulated during development, we employed hierarchical clustering. Each RSS/ADME gene received a score of + 1 for a given histone modification in a given tissue if it experienced a significant increase in abundance, or -1 if it experienced a significant decrease in abundance. Genes which experienced no change in that modification received a score of 0. We then performed hierarchical clustering within each tissue based upon these scores. We specified that five clusters be defined by the dendrogram for each tissue and identified loci that exemplified the pattern of changes in histone modifications. In the kidney, cluster 1 was characterized by loci like *Slc44a4* which gained the activating modifications while undergoing no change or a decrease in H3K27me3 (Fig. 6A, S1A, Table S4). Cluster 5 instead included loci like *Slc43a1* which gained H3K27me3 and tended to lose the activating modifications (Figure S1B). In the liver, cluster 1 contained genes like *Slc14a1* lost the activating modifications, whereas cluster 3 included genes like *Them4* which gained activating modifications without experiencing significant changes in the abundance of H3K27me3 (Fig. 6B, S2A and S2B, Table S4). In the brain, cluster 1 included genes like *Slc25a29* that gained activating modifications, whereas cluster 3 included genes like *Slc6a12* defined by deposition of H3K27me3 over development (Fig. 6C, S3A and S3B, Table S4). For each tissue, the dendrograms atop the heatmaps separated the activating modifications from H3K27me3, demonstrating that the activating modifications changed similarly under a distinct pattern of regulation versus the repressive modification. Proportional Venn diagrams (generated using DeepVenn [55, 56]) demonstrated that the subset of genes which underwent significant increases H3K4me3 overlapped with the subset of genes which experienced significant increases in the activating modifications H3K27ac and H3K9ac (Figure S4A). This effect was most pronounced in the liver, which exhibited the greatest overlap between subsets of genes that underwent developmental activation at the chromatin level. In contrast, genes which experienced a significant increase in H3K27me3 constituted a subset of RSS/ADME genes that was largely distinct from those that underwent deposition of activating modifications (Figure S4A). Genes that lost activating modifications also tended to lose them together (especially in the liver), and without experiencing loss of H3K27me3 (Figure S4B). These findings demonstrate that large subsets of RSS/ADME genes undergo activation at the level of chromatin as the kidney and liver mature into adulthood, whereas no similar process transpires in the brain. Indeed, the largest cluster of genes in the brain (cluster 4) were those which underwent no significant change in any of the histone modifications. In accordance, genes encoding the transcription factors *Hnf1a*, *Hnf4a*, *Ppara* and *Nr1h4* (encoding the farnesoid X receptor FXR), which are known to regulate the expression of RSS/ADME genes in the liver and kidney [5], underwent an obvious program of epigenetic activation in these organs that was not observed in the brain (Figure S5).

To determine whether these changes at the epigenetic level led to changes in the abundance of the corresponding transcripts, we examined publicly available RNAseq data from the ENCODE (Encyclopedia of DNA Elements) consortium database [39] by plotting the log_2_ ratio of transcript abundance in the adult versus embryonic organs within the identified clusters. Cluster 1 in the kidney, which gained activating histone modifications, also exhibited the highest overall ratio of transcript abundance (Fig. 6D). The kidney’s cluster 5, which became silenced at the chromatin level also exhibited the lowest average ratio of transcript abundance. In the liver’s cluster 3, which showed a clear signature of activation at the chromatin level, the ratio of transcript abundance was highest (Fig. 6D). Cluster 1 from the liver, on the other hand, lost activating modifications and had the lowest ratio of transcript abundance. Transcript levels remained largely unchanged during brain development across the 5 clusters, in agreement with the limited degree to which chromatin state within RSS/ADME genes was remodeled during neurodevelopment.

Finally, we performed hierarchical clustering across RSS/ADME genes on the combined data for all the modifications in all the tissues. The activating modifications H3K4me3, H3K27ac and H3K9ac clustered together according to the tissue in which they were detected (Fig. 6E). H3K27me3 instead resided within a cluster that contained data from all three organs. These findings demonstrate that the activating histone modifications are largely developmentally coregulated in these tissues, whereas regulation of the repressive modification H3K27me3 more similar across tissues.

### Comparative network analysis of chromatin state reveals tissue-specific hub genes

**Fig. 7:**
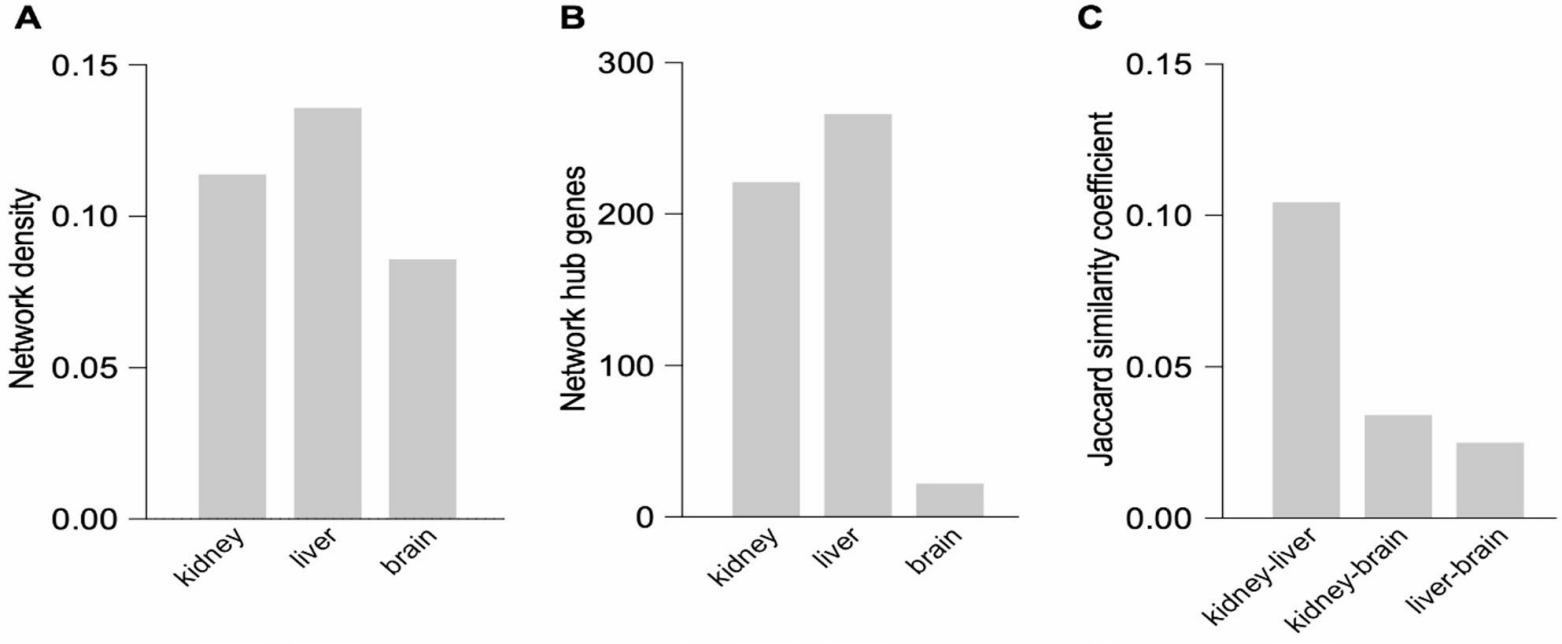
Network analysis of RSS/ADME genes demonstrates greater interconnectedness and overlap between non-neural organs. **A** Network density of log_2_ fold-change values (embryonic versus adult) in the four key regulatory histone modifications. **B** Number of network hub genes for each tissue. **C** Fraction of overlapping hub genes in pairwise comparisons

To capture the dynamics of histone modifications across RSS/ADME loci, we built weighted Spearman correlation networks for kidney, liver, and brain using developmental log_2_ fold changes computed for H3K4me3, H3K27ac, H3K9ac, and H3K27me3. By retaining only edges with Spearman ρ ≥ 0.9, we generated highly interconnected graphs in the liver (density 0.136) and kidney (density 0.112), which contrasted with a sparser network in the brain (density 0.086) (Fig. 7A). By applying an 80th-percentile cutoff on the weighted degree centrality metric, we identified 221 hub genes in the kidney and 266 in the liver, but only 22 in the brain (Fig. 7B, Table S5). Finally, we measured the overlap between the identified hub gene sets using Jaccard similarity coefficients [57]. The degree to which hub genes overlapped was much higher when comparing the kidney and liver, whereas very few hub genes from either non-neural organ overlapped with the hub genes identified in the brain (Fig. 7C).

Proportional Venn diagrams depicting the overlap between RSS/ADME genes that experienced increases in activating modifications demonstrated the largest overlap occurred when comparing the liver and kidney, which overlapped more with each other than either tissue did with brain (Figure S6A). Among RSS/ADME loci that lost activating modifications, the extent of overlap between liver and kidney was reduced relative to loci that gained activating modifications (Figure S6B). Interestingly, for H3K27me3 the overlap between liver and kidney was not much greater than the overlap of either tissue with the brain, as there was a great degree of overlap across all three tissues when it came to RSS/ADME genes that lost H3K27me3 (Figure S6C). These findings indicate that RSS/ADME loci are more extensively coregulated in the liver and kidney, organs that directly mediate interorgan communication, compared to the brain, which responds to but does not directly mediate RSS.

## Discussion

In this study, we examine for the first time how chromatin state within genes that regulate interorgan communication changes during the development of the mammalian liver and kidney [11, 12]. We also examine these genes in the brain, whose physiology depends upon proper regulation of blood-borne signals. We curated these 1034 protein coding genes from nodes in a published RSS network as well as lists of protein-coding genes known to play critical roles in RSS and/or ADME [4]. As shown in Fig. 1, this gene set consisted mainly of SLC and ABC transporters, Phase 1 and Phase 2 drug metabolizing enzymes, and nuclear receptors and transcription factors regulating these transporters and enzymes. We believe this represents represent a good, but by no means final, representation of the genes and proteins that regulate small molecule RSS.

Our examination of developmental changes in epigenetic regulation at RSS/ADME loci led to several insights. Across all three organs, the acetylated modification H3K27ac are more dynamic than the modifications H3K4me3 and H3K9ac within RSS/ADME genes. RSS/ADME genes in the kidney and liver are also more likely to experience a significant decline in H3K27me3 than a significant rise, demonstrating that derepression contributes to the acquisition of an activated gene expression state during liver and kidney development. Compared with non-RSS/ADME genes, genes regulating RSS/ADME in the liver and kidney are much more likely to undergo developmental deposition of activating histone modifications and/or loss of the repressive modification H3K27me3, demonstrating that these genes are subject to a specific regulatory logic. Network analysis of epigenetic data demonstrated that RSS/ADME genes are more highly interconnected in the kidney and liver (which harbor greater numbers of hub genes than the brain), demonstrating enhanced coordination during developmental chromatin reorganization.

Unbiased clustering according to changes in chromatin state led to the identification of a clear signature of epigenetic activation within subsets of RSS/ADME genes in the kidney and liver but not the brain. Importantly, these changes are accompanied by commensurate dynamism of the corresponding transcripts. Interestingly, among the 29 and 60 RSS/ADME genes in the kidney and liver, respectively, which exhibited the clearest signature of developmental activation (deposition of activating modifications accompanied by loss of H3K27me3 and transcriptional upregulation), 10 are common to both organs. Among these 10 genes, *Slc25a48*, *Slc16a12*, *Acot12* and *Pde8a* stand out as a reflection of the shared goal of regulating small molecules involved in metabolic communication, which includes substrates related to mitochondrial oxidative phosphorylation, fatty acids, and cyclic nucleotides [5, 23]. In the liver, activation of the genes *Cyp27a1* and *Cyp7b1* indicates regulation of bile acids, which have been extensively examined as key mediators of organ crosstalk and RSS [58]. Therefore our study complements prior literature that identified small molecules which must be autonomously handled to establish organismal autonomy. Future studies should address the extent to which epigenetic regulation drives the physiologic functions of these molecules to determine how the activated genes mediate handling of specific class of small molecules more generally, and what physiologic or developmental purposes these may serve [43, 46].

It will also be important to determine how epigenetic regulation is perturbed in diseases that impact RSS, or for which perturbation of RSS contributes as a pathogenic mechanism. For example, in chronic kidney disease (CKD), there is an accumulation of uremic toxins (e.g., indoxyl sulfate, TMAO), which are not easily removed through standard hemodialysis, and which cause progressive renal disease and cardiovascular morbidity [14]. Uremic toxins arise in the gut microbiome and then move into the circulation, whereupon they are taken up by the liver and modified before being eliminated by the kidney. Many RSS/ADME genes are involved in these processes, including SLC and ABC transporters and “drug” metabolizing enzymes [21–23]. Modifying the epigenetic landscape affecting these genes may prove an effective means for improving outcomes in CKD.

The physiological requirements between embryonic development and the postnatal period require an efficient system for small molecule communication to be established between organs. Our study indicates that many RSS/ADME genes, especially in the liver and kidney, become epigenetically activated during this period. This is not only relevant to the understanding of physiology after birth, but also to understanding the pathogenesis of several chronic diseases and the biological basis of postnatal pharmacokinetics [2].

## Conclusions

Tissue-specific patterns of developmental chromatin and epigenetic regulation occur within RSS/ADME genes in the mammalian liver, kidney and brain, a result which is generally compatible with the RSS Theory. Our findings have important implications for understanding both the establishment of interorgan small molecule communication as well as drug handling.

## Supplementary Information

Supplementary Material 1. Figure S1: Example loci from clusters of RSS/ADME genes identified in the kidney. (A) CUT&RUN tracks of H3K4me3, H3K27ac, H3K9ac, and H3K27me3 at the* Slc44a4* gene. Below each bigwig track is a BED file track showing peak regions called by the SEACR algorithm [31]. (B) CUT&RUN tracks at the* Slc43a1* locus. Figure S2: Example loci from clusters of RSS/ADME genes identified in the liver. (A) CUT&RUN tracks at the* Slc14a1* locus. (B) CUT&RUN tracks at the* Them4* locus. Figure S3: Example loci from clusters of RSS/ADME genes identified in the brain. (A) CUT&RUN at the* Slc25a29* gene. (B) CUT&RUN tracks at the* Slc6a12* gene. Figure S4: Activating histone modifications are coregulated at RSS/ADME loci. (A) Proportional Venn diagrams generated using DeepVenn [55,56] that reflect the overlap between loci which experience a significant increase in the abundance of each histone modification during the development of the indicated tissues. (B) Overlap between loci which experience a significant decrease in the abundance of each histone modification during tissue development. Figure S5: Key transcription factors driving RSS/ADME gene regulation undergo epigenetic activation in the kidney and liver. Normalized CUT&RUN tracks of H3K4me3, H3K27ac, H3K9ac and H3K27me3 in the embryonic and adult kidney, liver and brain at the* Hnf1a*,* Hnf4a*, Ppara and* Nr1h4* genes. Below each bigwig track is a BED file track showing peak regions called by the SEACR algorithm [31]. Figure S6: Activating histone modifications are coregulated at RSS/ADME loci. (A) Proportional Venn diagrams reflecting the overlap between subsets of RSS/ADME genes that undergo significant increases in activating histone modifications during the development of the indicated tissues. (B) Proportional Venn diagrams reflecting the overlap between subsets of RSS/ADME genes that undergo significant decreases in activating histone modifications during the development of the indicated tissues. (C) Similar analysis of H3K27me3.

Supplementary Material 2. Table S1: List of ADME genes and their categorization. Table S2: Quantitation of histone modifications. Table S3: Statistical analysis of CUT&RUN data. Table S4: Hierarchical clustering. Table S5: Hub genes.

## Data Availability

All raw sequencing files These are available from NCBI under the accession number GSE295997 at the link [https://www.ncbi.nlm.nih.gov/geo/query/acc.cgi? acc=GSE295997](https:/www.ncbi.nlm.nih.gov/geo/query/acc.cgi? acc=GSE295997) .

## Abbreviations

RSS: Remote sensing and signaling
ADME: Absorption, digestion, metabolism, excretion
SLC: Solute carrier
ABC: ATP-binding cassette
