# Supplementary figures and images for: Developmentally dynamic chromatin state at loci regulating organ crosstalk by remote sensing and signaling
Source: Epigenetics Chromatin. 2025 Dec 3;18:82. doi: 10.1186/s13072-025-00648-9 (PMC12720468; doi:10.1186/s13072-025-00648-9)

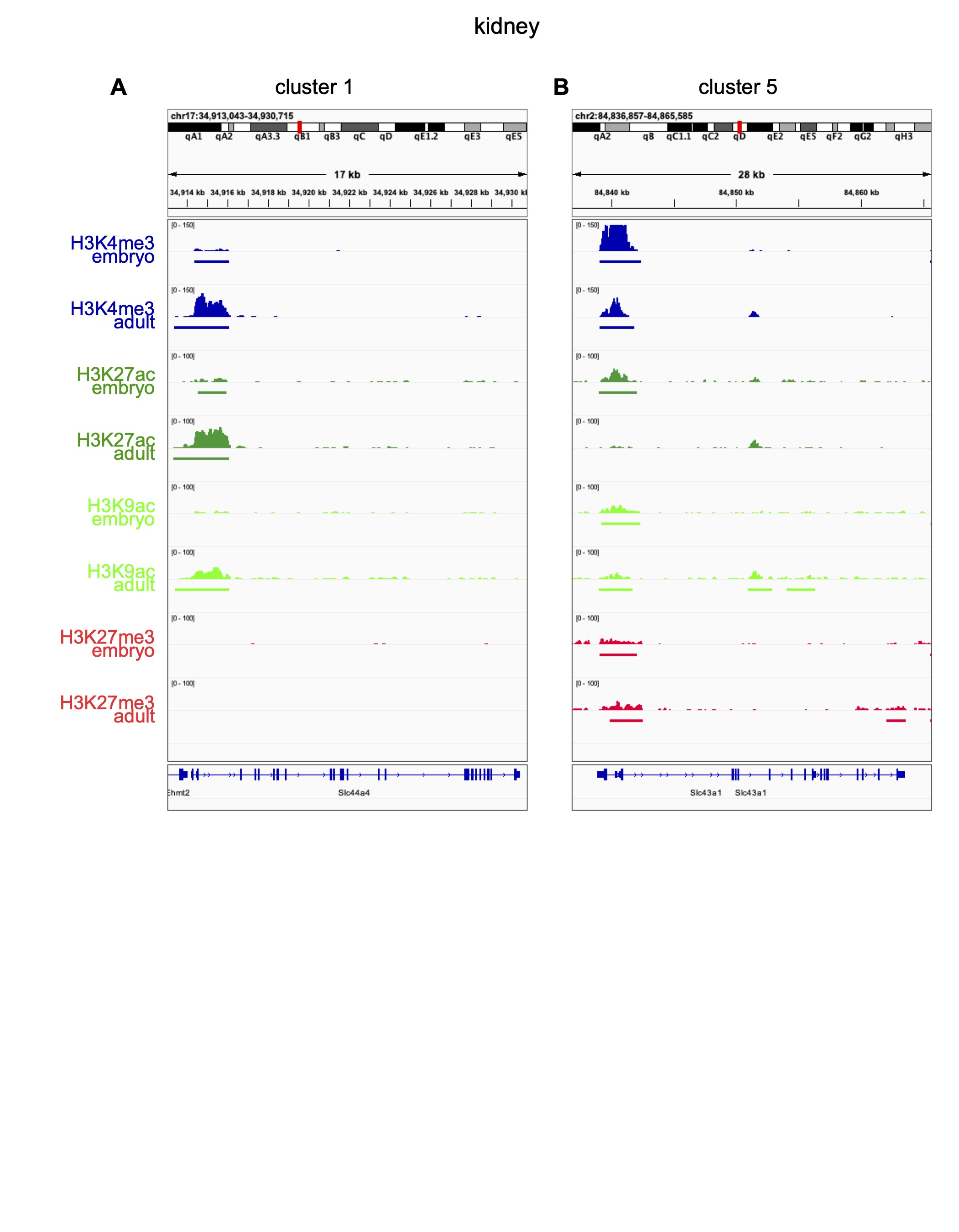

Supplement: Supplementary file 1 — Supplementary Material 1. Figure S1: Example loci from clusters of RSS/ADME genes identified in the kidney. (A) CUT&RUN tracks of H3K4me3, H3K27ac, H3K9ac, and H3K27me3 at the Slc44a4 gene. Below each bigwig track is a BED file track showing peak regions called by the SEACR algorithm [31]. (B) CUT&RUN tracks at the Slc43a1 locus. Figure S2: Example loci from clusters of RSS/ADME genes identified in the liver. (A) CUT&RUN tracks at the Slc14a1 locus. (B) CUT&RUN tracks at the Them4 locus. Figure S3: Example loci from clusters of RSS/ADME genes identified in the brain. (A) CUT&RUN at the Slc25a29 gene. (B) CUT&RUN tracks at the Slc6a12 gene. Figure S4: Activating histone modifications are coregulated at RSS/ADME loci. (A) Proportional Venn diagrams generated using DeepVenn [55,56] that reflect the overlap between loci which experience a significant increase in the abundance of each histone modification during the development of the indicated tissues. (B) Overlap between loci which experience a significant decrease in the abundance of each histone modification during tissue development. Figure S5: Key transcription factors driving RSS/ADME gene regulation undergo epigenetic activation in the kidney and liver. Normalized CUT&RUN tracks of H3K4me3, H3K27ac, H3K9ac and H3K27me3 in the embryonic and adult kidney, liver and brain at the Hnf1a, Hnf4a, Ppara and Nr1h4 genes. Below each bigwig track is a BED file track showing peak regions called by the SEACR algorithm [31]. Figure S6: Activating histone modifications are coregulated at RSS/ADME loci. (A) Proportional Venn diagrams reflecting the overlap between subsets of RSS/ADME genes that undergo significant increases in activating histone modifications during the development of the indicated tissues. (B) Proportional Venn diagrams reflecting the overlap between subsets of RSS/ADME genes that undergo significant decreases in activating histone modifications during the development of the indicated tissues. (C) Similar analy [file 13072_2025_648_MOESM1_ESM.zip › New folder/Figure S1 - kidney module genes.jpg]

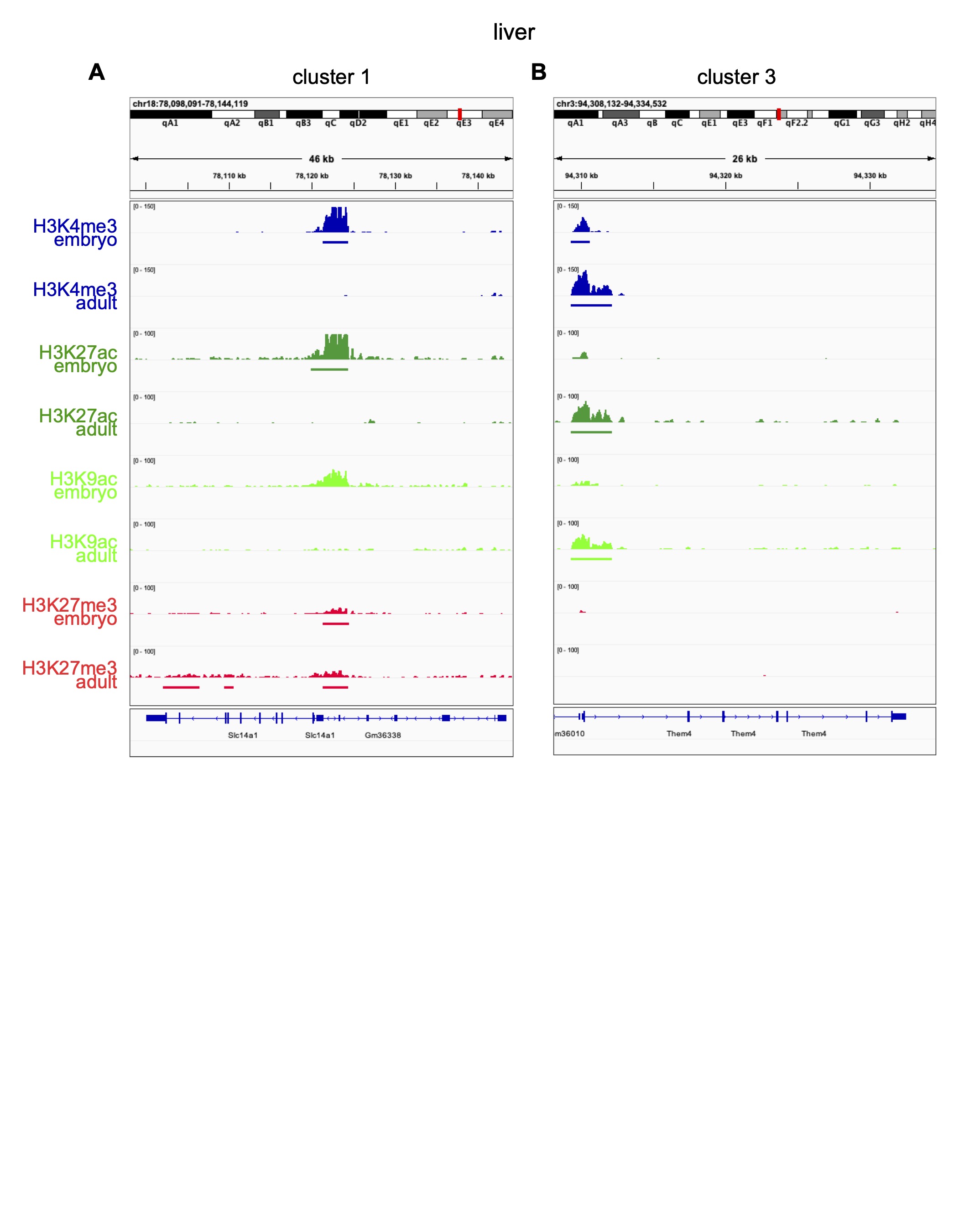

Supplement: Supplementary file 1 — Supplementary Material 1. Figure S1: Example loci from clusters of RSS/ADME genes identified in the kidney. (A) CUT&RUN tracks of H3K4me3, H3K27ac, H3K9ac, and H3K27me3 at the Slc44a4 gene. Below each bigwig track is a BED file track showing peak regions called by the SEACR algorithm [31]. (B) CUT&RUN tracks at the Slc43a1 locus. Figure S2: Example loci from clusters of RSS/ADME genes identified in the liver. (A) CUT&RUN tracks at the Slc14a1 locus. (B) CUT&RUN tracks at the Them4 locus. Figure S3: Example loci from clusters of RSS/ADME genes identified in the brain. (A) CUT&RUN at the Slc25a29 gene. (B) CUT&RUN tracks at the Slc6a12 gene. Figure S4: Activating histone modifications are coregulated at RSS/ADME loci. (A) Proportional Venn diagrams generated using DeepVenn [55,56] that reflect the overlap between loci which experience a significant increase in the abundance of each histone modification during the development of the indicated tissues. (B) Overlap between loci which experience a significant decrease in the abundance of each histone modification during tissue development. Figure S5: Key transcription factors driving RSS/ADME gene regulation undergo epigenetic activation in the kidney and liver. Normalized CUT&RUN tracks of H3K4me3, H3K27ac, H3K9ac and H3K27me3 in the embryonic and adult kidney, liver and brain at the Hnf1a, Hnf4a, Ppara and Nr1h4 genes. Below each bigwig track is a BED file track showing peak regions called by the SEACR algorithm [31]. Figure S6: Activating histone modifications are coregulated at RSS/ADME loci. (A) Proportional Venn diagrams reflecting the overlap between subsets of RSS/ADME genes that undergo significant increases in activating histone modifications during the development of the indicated tissues. (B) Proportional Venn diagrams reflecting the overlap between subsets of RSS/ADME genes that undergo significant decreases in activating histone modifications during the development of the indicated tissues. (C) Similar analy [file 13072_2025_648_MOESM1_ESM.zip › New folder/Figure S2 - liver module genes.jpg]

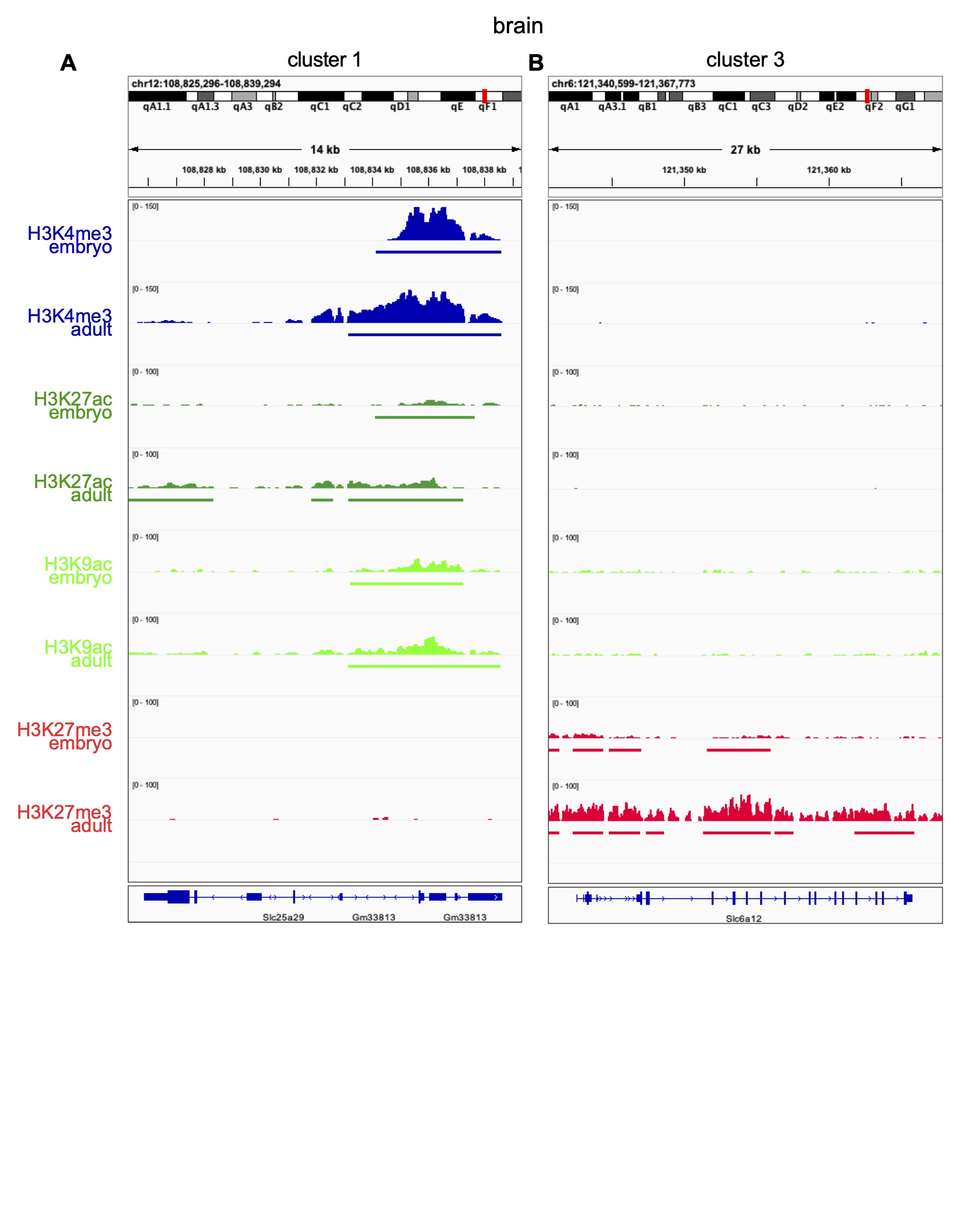

Supplement: Supplementary file 1 — Supplementary Material 1. Figure S1: Example loci from clusters of RSS/ADME genes identified in the kidney. (A) CUT&RUN tracks of H3K4me3, H3K27ac, H3K9ac, and H3K27me3 at the Slc44a4 gene. Below each bigwig track is a BED file track showing peak regions called by the SEACR algorithm [31]. (B) CUT&RUN tracks at the Slc43a1 locus. Figure S2: Example loci from clusters of RSS/ADME genes identified in the liver. (A) CUT&RUN tracks at the Slc14a1 locus. (B) CUT&RUN tracks at the Them4 locus. Figure S3: Example loci from clusters of RSS/ADME genes identified in the brain. (A) CUT&RUN at the Slc25a29 gene. (B) CUT&RUN tracks at the Slc6a12 gene. Figure S4: Activating histone modifications are coregulated at RSS/ADME loci. (A) Proportional Venn diagrams generated using DeepVenn [55,56] that reflect the overlap between loci which experience a significant increase in the abundance of each histone modification during the development of the indicated tissues. (B) Overlap between loci which experience a significant decrease in the abundance of each histone modification during tissue development. Figure S5: Key transcription factors driving RSS/ADME gene regulation undergo epigenetic activation in the kidney and liver. Normalized CUT&RUN tracks of H3K4me3, H3K27ac, H3K9ac and H3K27me3 in the embryonic and adult kidney, liver and brain at the Hnf1a, Hnf4a, Ppara and Nr1h4 genes. Below each bigwig track is a BED file track showing peak regions called by the SEACR algorithm [31]. Figure S6: Activating histone modifications are coregulated at RSS/ADME loci. (A) Proportional Venn diagrams reflecting the overlap between subsets of RSS/ADME genes that undergo significant increases in activating histone modifications during the development of the indicated tissues. (B) Proportional Venn diagrams reflecting the overlap between subsets of RSS/ADME genes that undergo significant decreases in activating histone modifications during the development of the indicated tissues. (C) Similar analy [file 13072_2025_648_MOESM1_ESM.zip › New folder/Figure S3 - brain module genes.jpg]

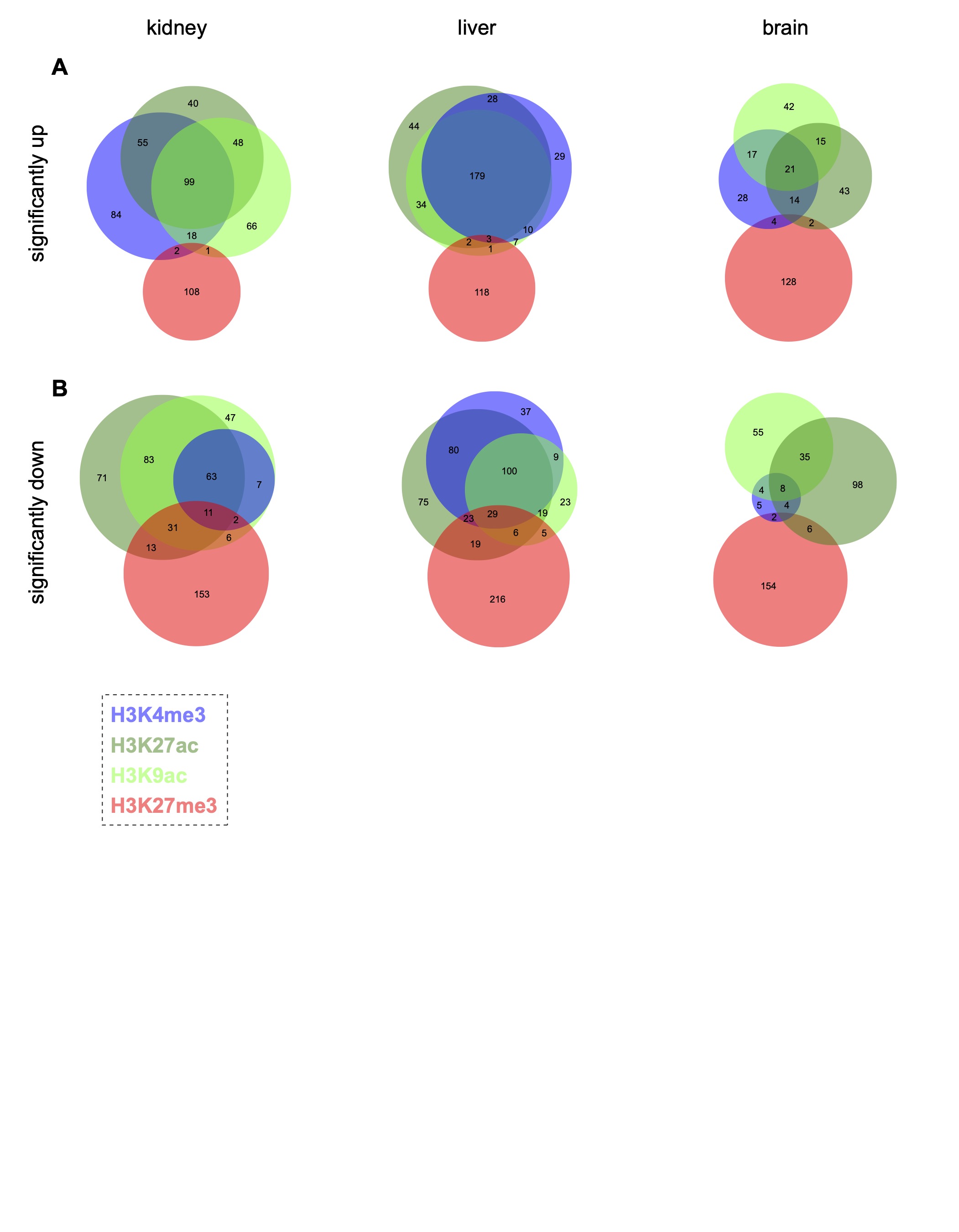

Supplement: Supplementary file 1 — Supplementary Material 1. Figure S1: Example loci from clusters of RSS/ADME genes identified in the kidney. (A) CUT&RUN tracks of H3K4me3, H3K27ac, H3K9ac, and H3K27me3 at the Slc44a4 gene. Below each bigwig track is a BED file track showing peak regions called by the SEACR algorithm [31]. (B) CUT&RUN tracks at the Slc43a1 locus. Figure S2: Example loci from clusters of RSS/ADME genes identified in the liver. (A) CUT&RUN tracks at the Slc14a1 locus. (B) CUT&RUN tracks at the Them4 locus. Figure S3: Example loci from clusters of RSS/ADME genes identified in the brain. (A) CUT&RUN at the Slc25a29 gene. (B) CUT&RUN tracks at the Slc6a12 gene. Figure S4: Activating histone modifications are coregulated at RSS/ADME loci. (A) Proportional Venn diagrams generated using DeepVenn [55,56] that reflect the overlap between loci which experience a significant increase in the abundance of each histone modification during the development of the indicated tissues. (B) Overlap between loci which experience a significant decrease in the abundance of each histone modification during tissue development. Figure S5: Key transcription factors driving RSS/ADME gene regulation undergo epigenetic activation in the kidney and liver. Normalized CUT&RUN tracks of H3K4me3, H3K27ac, H3K9ac and H3K27me3 in the embryonic and adult kidney, liver and brain at the Hnf1a, Hnf4a, Ppara and Nr1h4 genes. Below each bigwig track is a BED file track showing peak regions called by the SEACR algorithm [31]. Figure S6: Activating histone modifications are coregulated at RSS/ADME loci. (A) Proportional Venn diagrams reflecting the overlap between subsets of RSS/ADME genes that undergo significant increases in activating histone modifications during the development of the indicated tissues. (B) Proportional Venn diagrams reflecting the overlap between subsets of RSS/ADME genes that undergo significant decreases in activating histone modifications during the development of the indicated tissues. (C) Similar analy [file 13072_2025_648_MOESM1_ESM.zip › New folder/Figure S4 - Venn.jpg]

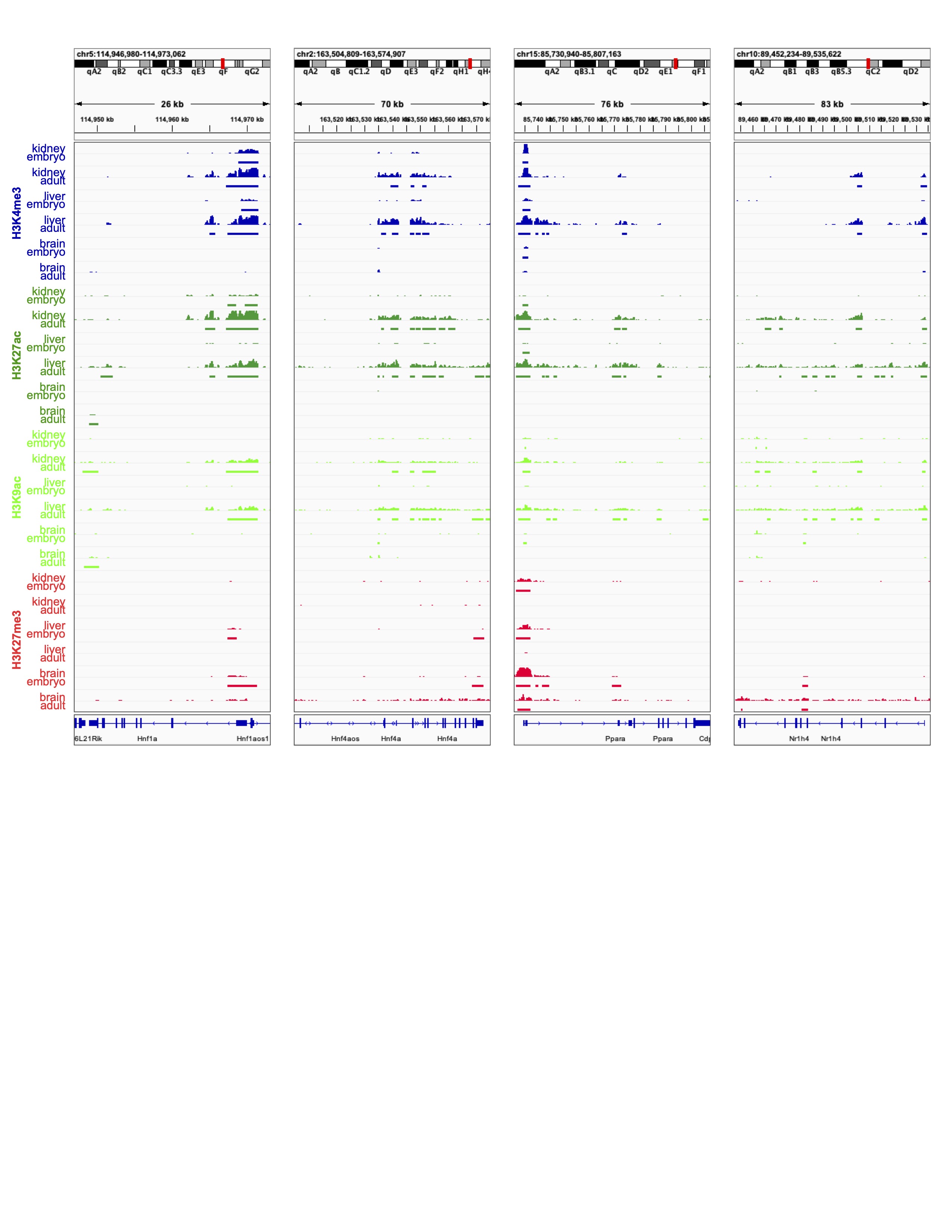

Supplement: Supplementary file 1 — Supplementary Material 1. Figure S1: Example loci from clusters of RSS/ADME genes identified in the kidney. (A) CUT&RUN tracks of H3K4me3, H3K27ac, H3K9ac, and H3K27me3 at the Slc44a4 gene. Below each bigwig track is a BED file track showing peak regions called by the SEACR algorithm [31]. (B) CUT&RUN tracks at the Slc43a1 locus. Figure S2: Example loci from clusters of RSS/ADME genes identified in the liver. (A) CUT&RUN tracks at the Slc14a1 locus. (B) CUT&RUN tracks at the Them4 locus. Figure S3: Example loci from clusters of RSS/ADME genes identified in the brain. (A) CUT&RUN at the Slc25a29 gene. (B) CUT&RUN tracks at the Slc6a12 gene. Figure S4: Activating histone modifications are coregulated at RSS/ADME loci. (A) Proportional Venn diagrams generated using DeepVenn [55,56] that reflect the overlap between loci which experience a significant increase in the abundance of each histone modification during the development of the indicated tissues. (B) Overlap between loci which experience a significant decrease in the abundance of each histone modification during tissue development. Figure S5: Key transcription factors driving RSS/ADME gene regulation undergo epigenetic activation in the kidney and liver. Normalized CUT&RUN tracks of H3K4me3, H3K27ac, H3K9ac and H3K27me3 in the embryonic and adult kidney, liver and brain at the Hnf1a, Hnf4a, Ppara and Nr1h4 genes. Below each bigwig track is a BED file track showing peak regions called by the SEACR algorithm [31]. Figure S6: Activating histone modifications are coregulated at RSS/ADME loci. (A) Proportional Venn diagrams reflecting the overlap between subsets of RSS/ADME genes that undergo significant increases in activating histone modifications during the development of the indicated tissues. (B) Proportional Venn diagrams reflecting the overlap between subsets of RSS/ADME genes that undergo significant decreases in activating histone modifications during the development of the indicated tissues. (C) Similar analy [file 13072_2025_648_MOESM1_ESM.zip › New folder/Figure S5 - TFs.jpg]

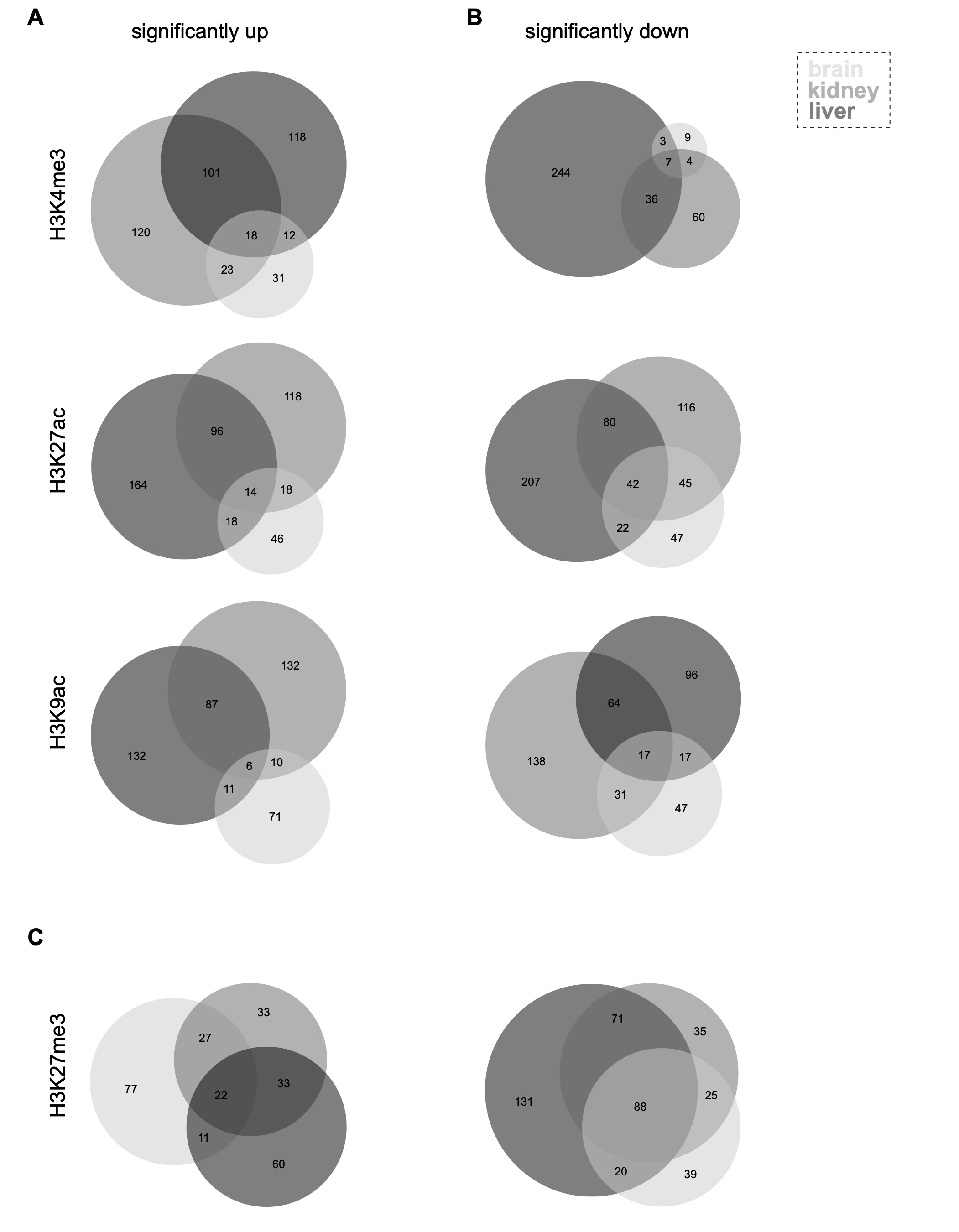

Supplement: Supplementary file 1 — Supplementary Material 1. Figure S1: Example loci from clusters of RSS/ADME genes identified in the kidney. (A) CUT&RUN tracks of H3K4me3, H3K27ac, H3K9ac, and H3K27me3 at the Slc44a4 gene. Below each bigwig track is a BED file track showing peak regions called by the SEACR algorithm [31]. (B) CUT&RUN tracks at the Slc43a1 locus. Figure S2: Example loci from clusters of RSS/ADME genes identified in the liver. (A) CUT&RUN tracks at the Slc14a1 locus. (B) CUT&RUN tracks at the Them4 locus. Figure S3: Example loci from clusters of RSS/ADME genes identified in the brain. (A) CUT&RUN at the Slc25a29 gene. (B) CUT&RUN tracks at the Slc6a12 gene. Figure S4: Activating histone modifications are coregulated at RSS/ADME loci. (A) Proportional Venn diagrams generated using DeepVenn [55,56] that reflect the overlap between loci which experience a significant increase in the abundance of each histone modification during the development of the indicated tissues. (B) Overlap between loci which experience a significant decrease in the abundance of each histone modification during tissue development. Figure S5: Key transcription factors driving RSS/ADME gene regulation undergo epigenetic activation in the kidney and liver. Normalized CUT&RUN tracks of H3K4me3, H3K27ac, H3K9ac and H3K27me3 in the embryonic and adult kidney, liver and brain at the Hnf1a, Hnf4a, Ppara and Nr1h4 genes. Below each bigwig track is a BED file track showing peak regions called by the SEACR algorithm [31]. Figure S6: Activating histone modifications are coregulated at RSS/ADME loci. (A) Proportional Venn diagrams reflecting the overlap between subsets of RSS/ADME genes that undergo significant increases in activating histone modifications during the development of the indicated tissues. (B) Proportional Venn diagrams reflecting the overlap between subsets of RSS/ADME genes that undergo significant decreases in activating histone modifications during the development of the indicated tissues. (C) Similar analy [file 13072_2025_648_MOESM1_ESM.zip › New folder/Figure S6 - Venn.jpg]
